# A highly mutagenised barley (*cv.* Golden Promise) TILLING population coupled with strategies for screening-by-sequencing

**DOI:** 10.1186/s13007-019-0486-9

**Published:** 2019-08-24

**Authors:** Miriam Schreiber, Abdellah Barakate, Nicola Uzrek, Malcolm Macaulay, Adeline Sourdille, Jenny Morris, Pete E. Hedley, Luke Ramsay, Robbie Waugh

**Affiliations:** 1 Cell and Molecular Sciences, The James Hutton Institute, Errol Road, Invergowrie, Dundee, DD2 5DA Scotland UK; 2 Division of Plant Sciences, University of Dundee at The James Hutton Institute, Invergowrie, Dundee, DD2 5DA Scotland UK; 3 School of Agriculture and Wine, University of Adelaide, Plant Genome Building, Waite Campus, Urrbrae, Adelaide, SA Australia

**Keywords:** Barley, Amplicon sequencing, Meiosis, Functional genomics, Exome capture, Recombination

## Abstract

**Background:**

We developed and characterised a highly mutagenised TILLING population of the barley (*Hordeum vulgare*) cultivar Golden Promise. Golden Promise is the ‘reference’ genotype for barley transformation and a primary objective of using this cultivar was to be able to genetically complement observed mutations directly in order to prove gene function. Importantly, a reference genome assembly of Golden Promise has also recently been developed. As our primary interest was to identify mutations in genes involved in meiosis and recombination, to characterise the population we focused on a set of 46 genes from the literature that are possible meiosis gene candidates.

**Results:**

Sequencing 20 plants from the population using whole exome capture revealed that the mutation density in this population is high (one mutation every 154 kb), and consequently even in this small number of plants we identified several interesting mutations. We also recorded some issues with seed availability and germination. We subsequently designed and applied a simple two-dimensional pooling strategy to identify mutations in varying numbers of specific target genes by Illumina short read pooled-amplicon sequencing and subsequent deconvolution. In parallel we assembled a collection of semi-sterile mutants from the population and used a custom exome capture array targeting the 46 candidate meiotic genes to identify potentially causal mutations.

**Conclusions:**

We developed a highly mutagenised barley TILLING population in the transformation competent cultivar Golden Promise. We used novel and cost-efficient screening approaches to successfully identify a broad range of potentially deleterious variants that were subsequently validated by Sanger sequencing. These resources combined with a high-quality genome reference sequence opens new possibilities for efficient functional gene validation.

## Background

Barley (2x = 2n = 14) is one of the world’s oldest and most important crops. While most of the harvested grain is used as animal feed, barley also underpins sectors of the food and particularly the drinks industry where it is a mainstay for the production of premium alcoholic beverages including beer and whisky. While high quality grain is needed to produce malted barley for the drinks industry (i.e. grain subjected to controlled germination then dried), grain failing to meet premium standards along with that grown purposely as high yielding lower quality grain, is directed towards animal feed. Traditional barley crop improvement for both of these end-use sectors has been in operation since the early twentieth century with formal breeding programs and research communities seeking out and embracing the use of wide genetic diversity. This has included variants induced by physical and/or chemical mutagenesis. Indeed, for previous research purposes many natural and induced morphological variants were used as the genetic markers that formed the basis of early genetic analyses in this species [1].

Mutation research and its application in barley is facilitated by it being a true diploid inbreeding crop, which allows rapid fixation and easy assessment of individual mutations and their subsequent exploitation in research and crop improvement. Importantly, mutation research in barley has had a significant practical impact. For example, the cultivar (*cv.*) Mari contains an induced mutation in *EARLY FLOWERING 3* (*HvELF3*) that was largely responsible for the northwards range extension of Scandinavian barley cultivation [2, 3]. Similarly, the barley *cv.* Golden Promise, a popular malting barley in the UK released in 1968 and still used by the Scotch Whisky industry today, is a γ-ray mutant of the *cv.* Maythorpe generated originally in 1956 [4]. It carries a loss-of-function mutation in the barley orthologue of rice *DENSE AND ERECT PANICLE 1* (*HvDEP1*), a heterotrimeric G-protein AGG3-type subunit encoding gene that positively regulates culm elongation and seed size [5].

While mutation research in barley was initially focused on the exploitation of variants that improved aspects of production or end-use quality, over the last 15 years the use of induced mutations has emerged as a key resource for gene discovery [6]. Using forward genetics approaches many genes, especially those conferring morphological or developmental phenotypes, have now been isolated [7–11]. In addition, Targeting Induced Local Lesions in Genomes (TILLING) [12] has become particularly powerful for gene validation studies and for exploring the phenotypic role of genes where no obvious visual phenotype of a given gene mutation can be predicted [13]. TILLING has been widely adopted and populations have been developed and used successfully for many crops, including tomato [14, 15], maize [16, 17], rice [18] and wheat [19, 20]. TILLING produces an allelic series, which is important for genes where a knock-out would be lethal but where impaired function may still allow the biological role of a gene to be studied. In barley, TILLING populations have been developed by several groups using several different cultivars [21–25]. One limitation of these available resources is that the parental cultivars used for TILLING population development are all recalcitrant to genetic transformation. Consequently, gene specific complementation assays, which offer a powerful validation strategy for quickly proving gene function, are generally not possible.

To overcome this, we have developed a heavily mutagenized EMS (ethyl methanesulfonate) TILLING population of *cv.* Golden Promise, the reference variety used across the barley research community for genetic transformation and functional genomics [26]. Our objective was to enable the possibility of using genetic complementation for validation of observed mutations in candidate genes that, amongst others, control the frequency and distribution of recombination in this large genome crop. As the transformation reference, Golden Promise is also the most efficient genotype for using emerging CRISPR–Cas9 based technologies [27, 28] which are becoming increasingly important in crop plant research. In parallel with establishing a TILLING resource, we have also recently completed the construction of a Golden Promise genome reference assembly (Schreiber et al., in prep). Together these will make Golden Promise an even more attractive choice for barley functional genomics research.

Meiotic recombination in barley exhibits a non-random pattern of events with most taking place at distal ends of the chromosomes while the centromeric region, that contains around 30% of the gene content, rarely recombines [29]. Our hypothesis is that mutations either in genes known to be involved in meiosis in different species (i.e. via reverse genetics) or those causing phenotypes indicative of perturbed meiosis (e.g. semi-sterility) will change the frequency or distribution of meiotic crossovers across the barley genome. This will in turn provide a better understanding of this fundamental process in a large genome crop, which we argue may be different, subtle or otherwise, from that observed in small genome models [30, 31]. The positive effect of mutations on recombination frequency and distribution in key meiotic genes has already been shown in *Arabidopsis* and other species [32–35].

Here, we evaluate and describe various features of our Golden Promise mutant population, including its development, mutation frequency and distribution, and demonstrate the use of different strategies to screen for mutations in a range of target genes, but in particular those involved in meiosis and recombination. We have exploited the fact that continuous improvements of next generation sequencing technologies are providing greater depths of high-quality data. Consequently, we focus exclusively on sequence-based mutation detection approaches. These allowed us to streamline the screening process by pooling plant DNA from multiple individuals instead of sequencing single lines [36]. We demonstrate the value of our resource by identifying multiple putatively deleterious mutations in 46 genes involved in meiosis and recombination and based on these results discuss whether sequencing barley mutant populations using e.g. exome sequencing, as successfully done in polyploid wheat [19], would be a feasible and valuable long-term strategy for the barley research community.

## Results

**Fig. 1:**
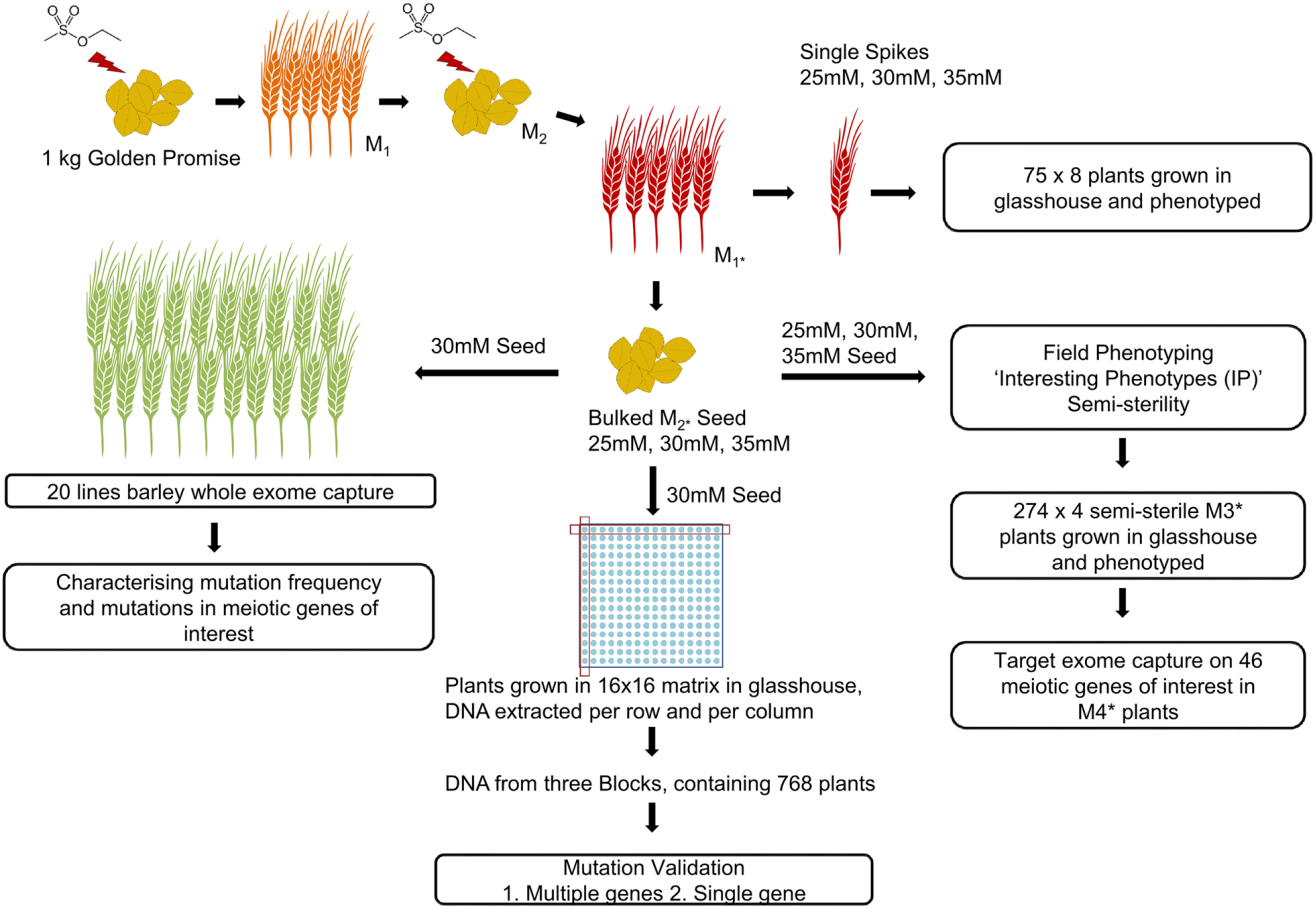
Experimental design of analysing the EMS TILLING population

We developed a twice mutagenised EMS population of barley *cv.* Golden Promise to both increase the mutational load and enable functional validation of candidate genes through routine transformation-based genetic complementation. We developed three streams of genetic materials: a structured mutant population for carrying out reverse genetics using a modified TILLING approach; a bulk seed resource for forward (and reverse) genetics screens and a phenotypically semi-sterile sub-population for research on meiosis and recombination (Fig. 1). Here we describe the general characterisation of this population and provide examples of screening for mutations.

### Characterising the population by whole exome capture sequencing

**Fig. 2:**
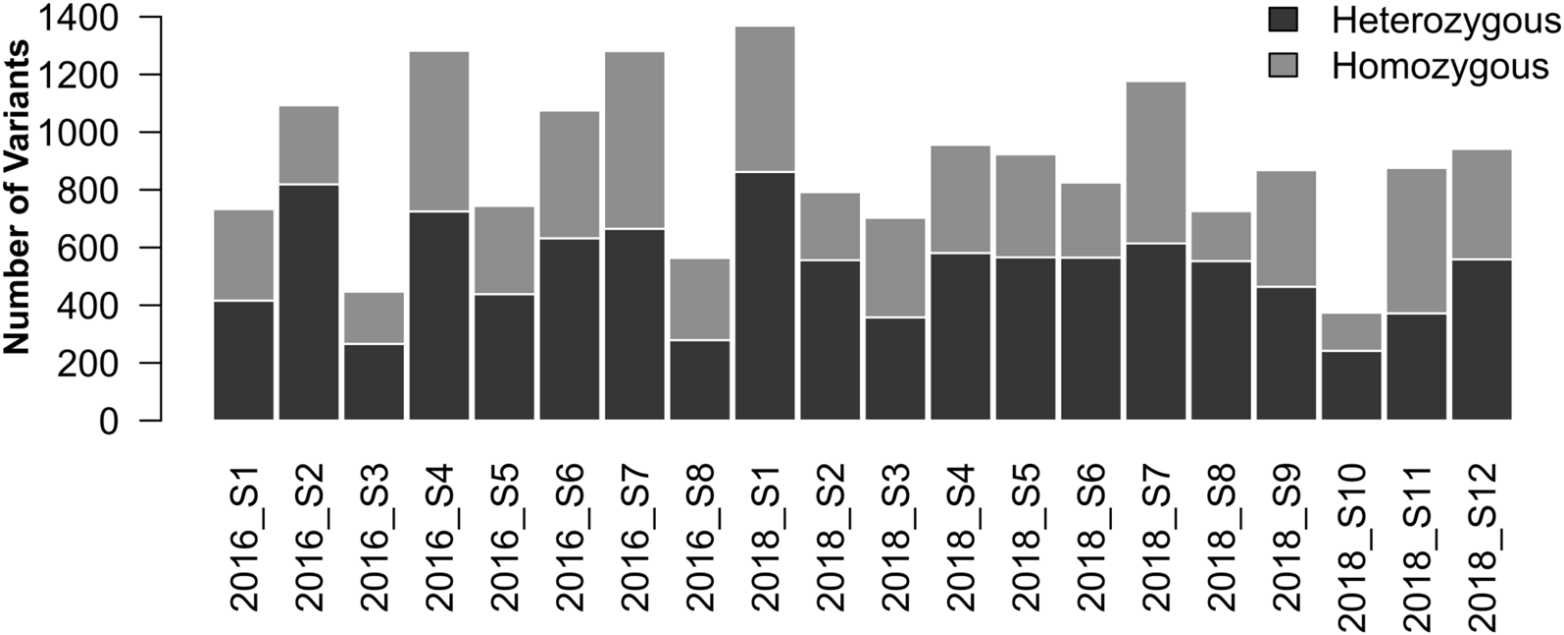
Variant distribution identified by barley whole exome capture per individual plants

We first used barley whole exome capture sequencing to estimate general characteristics of the Golden Promise mutant population. This capture has been estimated to cover 73.7% of the high-confidence and 40.7% of the low-confidence exon sequences annotated on the barley draft genome assembly [37, 38]. We choose random seeds from the bulk harvested M2* plants (see Fig. 1 and “Methods” section; star corresponding to the generation starting from the second mutagenesis) and extracted genomic DNA from leaves of 20 healthy looking M3* seedlings 2 weeks after germination and performed exome capture sequencing as described in “Methods” section. Sequencing reads were mapped against the barley *cv.* Golden Promise reference genome sequence (Schreiber et al. in prep) and the resulting variants filtered allowing only one variant in the twenty plants at any given position. We identified 17,818 single nucleotide polymorphisms (SNPs) with 7631 of those being on target (i.e. within the sequence covered by the capture array). The majority of identified variants were the expected G/C to A/T transitions (Additional file 1: Table S1). This included both heterozygous and homozygous mutations, which would be expected at this generation for a double mutagenized population. The identified mutations resulted in an average frequency of 1 per 154 kb, but as shown in Fig. 2 there were differences between individual plants. On average each plant contained 891 SNPs in the exome capture dataset with a distribution of 60% heterozygous and 40% homozygous.

To predict the effect of the observed mutations we first mapped the transcripts from the BaRTv1.0 reference transcript dataset [39] to our Golden Promise assembly to construct a personalised SNP-cured pseudo-reference transcriptome. After alignment to this personalised Golden Promise assembly we used SnpEff [40] for effect prediction. In total, 36.5% of the mutations were found within exons (Additional file 2: Figure S1) with 23.4% being missense (nonsynonymous) variants, 1.2% nonsense variants and 11.9% silent (synonymous) variants. From a total of 27.2% intron variants, 1.8% were found in splice sites. The remaining variants were found in intergenic regions, with 30% in the upstream or downstream interval of the transcripts, set by SnpEff as a 5 kb region. A complete table of the SnpEff results and effects on the individual transcripts listed by individual sample can be found in Additional file 3: Table S2. As our interest lay primarily in meiotic genes, we specifically checked our set of 46 candidate genes (Additional file 4: Table S3) for mutations. From these 20 plants, we identified 12 mutations in the coding regions of those genes plus additional mutations in intron or UTR region (Additional file 5: Table S4). We chose to validate two of these mutations (2016_S2-MRE11; 2016_S5-MUS81B) by growing four seeds from each sequenced parent plant. Three plants grew from plant 2016_S2 and three from 2016_S5. Sanger sequencing showed that all plants carried the identified mutations.

### Pooled amplicon sequencing of multiple target genes

While whole barley exome capture sequencing proved useful for gaining an impression of overall mutation frequency, type and distribution, we chose to explore the potential of cost-effective amplicon sequencing to screen for multiple mutations in a small genomic space. We adopted a DNA pooling strategy based on the 16 × 16 plant matrix described in “Methods” section (i.e. screening 256 plants twice in 32 DNA samples). We first chose to amplify genomic targets covering 400 bp regions in 10 barley meiotic genes. The 400 bp fragment size (except an *HvFANCM* fragment of 479 bp) was chosen both for compatibility with Illumina MiSeq paired-end 2 × 250 sequencing and to facilitate efficient multi-plex PCR amplification of all 10 fragments in each pooled DNA sample. After sequencing the reads were checked for quality, with 87.35% of the bases equal to or above the phred quality score of 30. All reads were then mapped to the respective gene sequences under the assumption that the 2D pooling would remove false positive variants originating from lower quality reads. We observed that two libraries appear to have failed as Block 1 Column 12 and Block 1 Row N had only 320 and 566 reads (while average reads per library were 108,000 reads) and visual inspection of the Bam files in Tablet [41] and target coverage analysis in Picard (v.2.18.4) [42] revealed that one gene, *HvFIGL1*, was only covered by 0.8% of the reads. Variant calling was done using Freebayes (v.0.9.18) [43] taking pool depth [16] and potential heterozygosity [2] into account by reducing the accepted minimum fraction of the alternate allele to 0.02 (i.e. 1:50 opposed to 1:32). Using the intrinsic features of the pooling strategy to remove random variants, we only kept those which occurred twice per block, once in a row and once in a column. We identified a total of 17 mutations meeting these relatively strict criteria, with three occurring twice (Additional file 6: Table S5). Seeds from mutant plants were then identified, and four seeds from each sourced for validation. However, of the original 17 plants, three were sterile with no seed harvested, and for four lines all seeds failed to germinate leaving ten that were possible to validate. For all ten, the same mutations, homozygous or heterozygous, were observed in the segregating progenies by Sanger sequencing.

### Pooled amplicon sequencing of a single target gene, HvMet1A

**Fig. 3:**
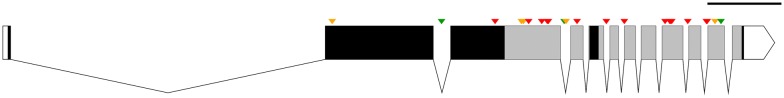
Locations of *HvMet1A* variants. Conserved domains are shown in grey, first the BAH domain (Bromo Adjacent Homology domain), followed by the Cytosine-C5 specific DNA methyltransferase domain. Identified variants are shown by arrows, red arrows highlight nonsynonymous variants, orange synonymous and green intron variants. A detailed summary of the variants is given in Additional file 7: Table S6. The size bar represents 1 kb (Figure was generated using http://wormweb.org/exonintron)

We next used amplicon sequencing to screen multiple regions tiled across *DNA* (*cytosine*-*5*) *methyltransferase 1* (*HvMET1*A), a well described gene involved in DNA-methylation. Thirteen regions of 400 bp length were chosen that covered two BAH domains in exons 3–5, a large C-5 cytosine DNA-methyltransferase domain on exons 5–12, and most of the splice junctions. After sequencing, the reads were checked for quality, which revealed that 80.5% were equal or above the phred quality score of 30. The same bioinformatics pipeline as above identified 30 mutations (Additional file 7: Table S6). As the original plants came from a bulk harvest of the previous generation, we could not assume categorically that all individuals were unique. A C2618T variant was identified in five plants, and three variants C2614T, G3977A and C4295T occurred in two plants. Thus, from the original 30 mutations, 23 independent variants were retained (Fig. 3) and classified as three intron mutations, five synonymous mutations and 15 nonsynonymous mutations. Most of the exon mutations were in the targeted ‘conserved-domains’ of *HvMET1A*. For the nonsynonymous mutations we calculated the PROVEAN score, the smaller the value the higher the confidence that this mutation might be deleterious for the protein function. This highlighted four different variants with values below -7 which are of interest for future experiments. In this case, mutation validation was only conducted for genomic DNA plate 1. Once again, two of the original source plants turned out to be sterile and for one line only three plants grew, all of which had the wild type allele. For the remaining plants all mutations were validated by PCR sequencing (Additional file 7: Table S6).

### General phenotypes within the population

Given the attrition rate we observed in seed or plant viability in these previous experiments we decided to quantify the effect of the high mutational load in the population by scoring obvious developmental phenotypes in M3* plant families. We considered this important because we expect that mutations in our prioritised meiotic genes may affect fertility or seed viability. We selected 75 random hand-harvested spikes from the field grown M2* plants and grew 8 seeds from each in plant trays in the glasshouse. 70% of the seed germinated (compared to 90% from wild type), most likely reflecting the mutational load. Early phenotyping showed segregation of numerous chlorophyll and albino phenotypes (Table 1). Further phenotypes included grass-like or bushy plants, thin or necrotic leaves and plants with dying leaf-margins. 30.3% of the plants showed no obvious phenotype throughout the growing period. At maturity, plants were screened for spike morphology and height. Of the mature plants 72% appeared to be fully fertile while the remainder were either completely or semi-sterile. 18 plants showed a pronounced dwarf phenotype.

**Table 1:** Phenotyping results of M2* plants

Categories	Phenotype	Number of mutants	Homozygous	Segregating
Spike morphology	Sterile	11	0	6
	Semi-sterile	98	12	11
	Dense	19	2	3
	Short	13	2	2
	Intermedium	1	0	1
	Long	4	1	0
Plant height	Dwarf	18	2	9
Chlorophyll	Albino	3	0	3
	Striata	1	0	1
	Yellow	11	1	3
Leaves	Necrotic	5	0	2
	Bushy	2	0	2
	Thin	18	2	13
	Dying-margins	8	1	1
Remaining	No ears	9	1	5
	Reduced tillers	11	1	5
	Late flowering	15	0	9
	Erect	32	3	10
	Grass-like	6	0	5

### Screening for semi-sterile lines

An established approach to study meiotic recombination is to analyse plants which show a semi-sterile phenotype as a proportion of these are expected to be impaired in meiosis. Seeds from all three M2* bulks (25 mM, 30 mM and 35 mM EMS concentration) were sown in the field in spring 2017. From these field plots we collected 274 semi-sterile M3* spikes. We planted four seed from each spike in the glass house. 15% of the seed did not germinate, 6% of plants died and 5% did not produce any fertile ears. The remaining plants were grown to maturity and scored for semi-sterility. 85% of the lines were either semi-sterile/sterile or segregating for this phenotype. Four lines were completely sterile, and three lines were identified as wild type (Table 2). All 239 remaining semi-sterile lines (segregating and/or already homozygous for this phenotype) were taken forward. Two seed for each line were grown again in the glass house. Of these, 179 lines germinated and were screened for mutations in 46 potential meiotic candidate genes using a custom designed MYbaits target exome capture (see “Methods” section). In total 98 mutations in the target genes were identified in 64 individual plants (Additional file 8: Table S7). 3 were nonsense mutations which introduced premature stop codons in three different genes. In addition, we identified 52 nonsynonymous, 21 synonymous variants and 22 variants in introns, a similar distribution to the whole barley exome capture results. Again, the PROVEAN score was calculated for each of the nonsynonymous variants to predict the deleterious effect of the variants. This highlighted six different mutations in five different genes that are predicted with high confidence to be deleterious of a PROVEAN score below -6.

**Table 2:** Phenotyping of semi-sterile M3* plants

Phenotype	Segregating	Homozygous	Total
Sterile	36	4	40
Semi-sterile	56	183	239
No ears	24	6	30
WT	15	3	18

## Discussion

TILLING is a powerful approach for reverse genetics and for the validation of candidate genes in gene discovery projects, in particular because it reveals an allelic series that can conclusively prove gene function [12]. TILLING populations can of course also be used for forward genetics, either for visible morphological or developmental phenotypes or for scoring phenotypic behaviour after the application of specific mutant screens. In many crop plants the use of mutants has largely and successfully focused on the former. However, advances in next generation sequencing technologies for high throughput genome characterisation has radically changed the value of TILLING. For example, the recent publication and release of exome capture sequences from populations of highly EMS mutagenized tetraploid and hexaploid wheat, has provided gene level induced variant information online to an entire research community, providing an immensely powerful resource in an important global crop [19].

Here we used whole exome capture, targeted exome capture and two-dimensional amplicon sequencing to characterise a TILLING population of the barley cultivar Golden Promise. This proved to be informative for both simultaneously identifying mutations of interest and accurately estimating the overall mutation frequency in the population. EMS, as used here, mainly causes G to A and C to T changes by alkylation of guanine which causes mispairing with thymine in the replication cycle. We therefore expected most of the mutations to be G/C to A/T transitions and our results showed that 79.4% fell into this category. The remaining mutations were 8.1% A/T to T/A, 6.8% G/C to T/A, 3.9% A/T to G/C and the remaining 1.8% to other transition/transversion events (Additional file 1: Table S1). As most previous reports have only looked at a small subset of genes it is difficult to compare these results to other barley resources [21, 22, 25]. The frequency is similar to that found in HvHox1 where 25 from 31 mutations were G/C to A/T transitions [22]. It is also similar to the recent results of Szurman-Zubrzycka et al. [44] who screened 32 genes and found 88% G/C to A/T transitions in the barley cultivar Sebastian. Keeping in mind that they used with NaN3/MNU combination a different chemical which is also known to mainly cause the above highlighted transitions but acts differently [24]. While the recently published wheat results show a 99% of G/C to A/T transition [19], other crops like rice [18] and tomato [14] have shown results more similar to barley. We believe that many of the unexpected mutations may have arisen through outcrossing, an unavoidable consequence of multiplying so many plants in a barley experimental nursery. While we rigorously removed lines that did not resemble Golden Promise phenotypically (e.g. they were unusually tall, had long awns or a lax spike) SNP genotyping of a small subset of the Golden Promise Mutants did reveal a low level of outcrossing in the population. It is also possible that genome wide sequence-based characterisation is much more sensitive and representative of overall mutation types and frequencies. Previous studies have generally used relatively insensitive heteroduplex *Cel1* assays focused on exon regions while we assessed intergenic and intronic variants. In wheat Henry et al. [45] found a strong EMS mutation site bias for an RGC motif (with R being either G or A) as a preferred target site, suggesting that both DNA sequence and GC content may explain why more mutations are found in some genes compared to others. In our whole exome capture experiment using only 20 plants, we found that the mutation frequency differed between individual plants and some genes were richer in mutations to others. Focusing specifically on the 46 potential meiotic genes of interest we identified 12 mutations in 8 different genes (Additional file 5: Table S4).

The Golden Promise TILLING population showed a frequency of 1 mutation every 154 kb. To put that into perspective, if a gene of interest is 2 kb in length one would need to screen around 80 plants on average to identify a single mutation. In comparison to other barley TILLING populations this represents one of the highest mutation loads for this diploid genome, with our phenotypic data suggesting that this is already at the border of what is possible without severely impairing fertility and vitality of the next generation [46]. In comparison, polyploid organisms such as hexaploid wheat with around 1 mutation every 40 kb [19, 20, 47], can tolerate higher mutation loads due to gene redundancy. There are two major downsides associated with such a high mutational load in barley. First, we found issues with seed viability in the M3* and to address this, we are currently advancing the population through single seed descent to eradicate severely compromise lines and maximise viability. This will inevitably result in the removal of a subset of lines. Second, multiple rounds of backcrossing may be required to remove secondary mutations, though our original idea was that this may be overcome by direct transgenic complementation. On the positive side, the chance of revealing multiple independent mutations in a single gene in a reasonably sized population is high, with multiple independent alleles providing direct support for association with a phenotype, thus avoiding the need for multiple backcrosses and allowing immediate phenotypic characterisation.

In our first proof of principle study we successfully used amplicon sequencing to identify mutations in 400 bp fragments selected across 10 genes. We identified multiple different mutations which could be validated in the subsequent generation. Although the fraction of the alternative allele in comparison to the reference allele is quite low in the analysis (for a heterozygous mutation below 0.03 and for a homozygous mutation below 0.06), with sufficient sequence depth it was possible to predict the zygosity correctly. However, this experiment also revealed some pitfalls. The first was the challenge of robust and representative PCR multiplexing. Individual genes were not monitored during the process and this resulted in the underrepresentation of one fragment (*HvFIGL1*) in comparison to the others. The second was that sequencing the libraries is not necessarily equal. In our case, two pools Block1 Column12 and Block1 Row N contained almost no reads. This already removes the potential for detecting mutations in 32 plants in this block. In our second amplicon sequencing experiment the approach was changed to detect mutations in the exons and splice junctions of barley *HvMET1A* gene. In *Arabidopsis thaliana* and rice (*Oryza sativa*), mutants in *AtMET1* and *OsMet1*–*2* are characterised by a wide range of epigenetic changes and a highly hypomethylated genome [48–50]. This results in delayed development, increased sterility and lethality, as well as an increased number of crossing overs in the centromere proximal region [51–53]. This makes it a potentially important gene for meiosis research in barley and thus our gene of choice for functional characterisation. We screened the whole population of 3072 plants for mutations within this gene. This time, a few samples of plate 1 were monitored using qPCR to confirm equal amplification of each individual PCR fragments across *HvMET1A* gene at the end of multiplex-PCR reactions. This highlighted consistent amplification across all primer pairs. Using this approach, we identified 30 mutations and validated a subset in the progeny plants. Pooled amplicon sequencing therefore allowed for the efficient and rapid identification of mutations in this particular target gene and is an approach we have now used for other genes of interest.

We have previously shown that studying semi-sterile desynaptic mutants can lead to new insights into recombination in barley [54]. Thus, we were interested in identifying newly-induced semi-sterile mutants for exploration in our research program and to supplement the classical desynaptic mutant collection we have available [55]. In our first round of phenotypic screening in the field we identified 274 plants with a semi-sterile phenotype. As this phenotype can also be influenced by environmental factors (like temperature) [56] and screening is largely subjective, we grew seeds from each spike in the glass house to confirm the phenotype. The lines in which the phenotype was confirmed were screened by targeted exome capture sequencing which identified a large number (98) of variants in potential meiotic genes. If we consider only the identified nonsynonymous and nonsense variants, there is the possibility that we have identified the causal genes/mutations behind 42 semi-sterile plants.

## Conclusion

We developed a highly mutagenised population of the transformation reference barley *cv.* Golden Promise by mutagenising twice with EMS. Characterising the population by exome sequencing revealed a mutation frequency of 1:154 kb, considerably higher than that reported for other barley TLLING populations. To exploit the population we developed and applied a range of sequence-based approaches for targeted mutation discovery in either single or multiple genes, and demonstrate the utility of the resource by identifying predicted deleterious mutations in 46 genes involved in meiosis and recombination. Given the high mutational load, we also observed higher rates of lethality than typically found in similar resources. We are therefore currently advancing the population through two rounds of single seed descent. The resulting seed from this advanced population will be made available to the community for screening. Alternatively, potential users are welcome to personally screen the M2* pools in the host laboratory. Were there no financial constraints, we believe it would be valuable to develop a genome wide database of induced mutations linked to a seed resource, like that now available for wheat [19]. This could be developed either by systematic whole barley exome capture sequencing of individual or multiple mutant populations or, as prices continue to decline, whole genome shotgun sequencing. This will almost certainly come down to a financial rather than a scientific decision.

## Methods

### Plant material

Approximately 20,000 barley (*Hordeum vulgare cv.* Golden Promise) seeds (1 kg) (M0) were mutagenized in 25 mM ethyl methanesulfonate (EMS) as described previously [21]. M1 plants were grown in the field in Dundee, Scotland in 2014. A visual inspection of the population during the growing season suggested a lower than expected rate of induced mutant phenotypes (e.g. chlorophyll mutants). As Golden Promise has a very distinct morphology (erect, short and stiff straw) rogue plants were easily and routinely identified and eradicated from the M1 plots. Seed from the M1 was bulk harvested and approximately 20,000 seed from this population re-mutagenised in 2015 using 25 mM, 30 mM and 35 mM EMS as before and separately grown in thinly sown field plots. We call these M1* plants (experimental setup is highlighted in Fig. 1). For each treatment approximately 4000 single spikes harboring M2* seeds were harvested by hand and the remaining seed from each treatment was bulk harvested. Approximately 15 kg of bulk seed from each treatment was retained. Random individual seeds from the 30 mM EMS M2* seed bulk, were used to grow M2* plants for barley whole exome capture and amplicon sequencing and four grain from each of 75 randomly chosen single spikes used for phenotyping. All plants were grown in the glasshouse under 16 h days at 20 °C (nominal) and 8 h nights at 15 °C (nominal). For phenotypic selection of semi-sterile mutants and other interesting inflorescence phenotypes, seeds from all three bulk harvested samples (M2* plants) were grown in the field in 2017 and mutants identified by visual inspection throughout the growing season. Kill rate was visually scored in these M2* plants (but not accurately quantified) in the field and was proportional to the concentration of EMS (35 mM > 30 mM > 25 mM).

For the development of our TILLING population, individual M2* seeds from the 30 mM EMS mutagenesis were potted in batches in a glasshouse in square 8 cM pots in twelve 16 × 16 pot arrays (3072 individual plants). Approximately 6–8 days after planting, dead (no germination), albino and weak plants (roughly 10–15% of the planted seed) were removed and replaced with healthy plants resulting in complete 16 × 16 row by column arrays. At the two-leaf stage four 1 cm leaf segments were cut from each plant: one was pooled with segments from the 15 other plants in the column orientation, one with segments from 15 other plants in the row. The remaining two segments were similarly pooled and kept frozen as a back-up. Genomic DNA was extracted from these pooled samples using DNeasy^®^ Plant Maxi Kit (Qiagen Gmbh, Hilden, Germany) and quantified with Quant-iT™ PicoGreen dsDNA Assay Kit (ThermoFisher Scientific, Waltham, MA, USA). All plants were then grown to maturity and seeds collected, indexed and archived.

### Barley whole exome capture

To evaluate the mutation frequency in the population, we used the barley whole exome capture sequencing approach (which can be obtained from Roche NimbleGen, Basel, Switzerland) [38]. A total of 20 seedlings grown from two separate batches of 8 and 12 individual 30 mM EMS M2* bulk seeds were used.

#### DNA extraction and library preparation

Leaf tissue of 10-day old plants was harvested and a Qiagen DNeasy Plant Mini Kit (Qiagen Gmbh) was used to extract DNA. DNA concentration was determined using a Qubit Fluorometer (ThermoFisher Scientific) and diluted to 2 ng/µl in 10 mM Tris–HCl pH9 buffer. The barley whole genome exome capture components were obtained from Roche NimbleGen (SeqCap EZ Developer probe pool design 120426_Barley_BEC_D04). Sample libraries were prepared following a plant-modified version of the NimbleGen SeqCap EZ Library User Guide v5.1 which is detailed below.

#### Sample library preparation

53 µl of 2 ng/µl DNA was fragmented using a Covaris M220 (Covaris, Inc., Woburn, MA, USA) aiming for a size range of 180–220 bp (50 W peak incident power, 20% duty factor, 200 cycles per burst, 280 s duration) then quality and concentration checked on a Bioanalyzer 2100 (Agilent Technologies, Santa Clara, CA, USA). Sample library preparation was done using the appropriate KAPA kit (KAPA Library Preparation Kit for Illumina, Roche). The Roche adapter index set A was used (SeqCap Adapter Kit A 96, Roche). 20 µl of the sample library was amplified in a pre-capture LM-PCR (ligation-mediated PCR, 50 µl; SeqCap EZ Accessory Kit v2, Roche; TS-PCR Oligo 1: AATGATACGGCGACCACCGAGA; TS-PCR Oligo 2: CAAGCAGAAGACGGCATACGAG; KAPA Hifi Hotstart; Ready mix 25 µl; Oligo mix 5 µl; program: 98 °C for 45 s, 9 cycles of 15 s at 98 °C, 30 s at 60 °C and 30 s at 72 °C, followed by 1 min at 72 °C). Libraries were cleaned, and concentrations quantified on a NanoDrop (ThermoFisher Scientific). Quality was again confirmed on the Bioanalyzer, aiming for a library with fragments in the range of 250–500 bp.

#### Hybridization and exome capture

Both libraries of 8- and 12-plant batches were pooled independently to a total of 1 µg DNA for further steps in library preparation. Hybridization enhancing (HE) oligos were mixed so that the resulting multiplexing pool contained equal amounts of the SeqCap HE Universal Oligo 1 (50%) and the mixture of appropriate SeqCap HE Index Oligos (50%). 10 µl SeqCap EZ Developer Reagent (Roche) was mixed in one tube with 1 µg of the Multiplex DNA Sample Library and a total of 2000 pmol of the Multiplex HE Oligo pool. The mixture was dried down then 7.5 µl of SC Hybridization Buffer and 3 µl of SC Hybridization Component A were added (SeqCap Hybridization and Wash Kit, Roche). The tube was vortexed for 20 s and then centrifuged for 10 s at 16,000*g*. After a denaturation step on the heat block at 95 °C for 10 min the sample was centrifuged and then added to a 0.2 ml PCR tube containing 4.5 µl SeqCap Exome Library. The sample was incubated in a thermocycler at 47 °C for 16–20 h.

#### Washing and recovering captured multiplexed DNA sample

The SeqCap capture beads (SeqCap Pure Capture Bead Kit, Roche) were warmed to room temperature and cleaned in two wash steps (first with 200 µl Bead Wash Buffer, second with 100 µl Bead Wash Buffer). Hybridization samples were quickly transferred to still-wet capture beads, mixed by pipetting up and down and left in the thermocycler for 45 min at 47 °C. Every 15 min the sample was vortexed for 3 s. Beads were washed to remove unbound fragments using the following steps: 100 µl of Wash Buffer I were added to the tube still in the thermocycler at 47 °C. The sample was vortexed for 10 s and the buffer removed. This was followed by twice adding 200 µl Stringent Wash Buffer, each time incubated at 47 °C for 5 min and then removed. The next three wash steps with Wash Buffer I, Wash Buffer II and Wash Buffer III were done at room temperature. Consecutively 200 µl of the buffer were added, vortexed for 2 min (Wash Buffer I), 1 min (Wash Buffer II), 30 s (Wash Buffer III) and each time removed. The beads were resuspended in 50 µl PCR-grade water and stored on ice. A post-capture LM-PCR was done to amplify the bead bound library (Identical to pre-capture LM PCR, except the number of cycles was increased to 14). Agencourt AMPure XP Beads were used to clean up the library which was then resuspended in 10 mM Tris–Cl pH8. 50 µl of supernatant which contains the amplified sample library were transferred into a new tube. A NanoDrop was used to determine DNA concentration and a Bioanalyzer to check for the correct fragment size of the final libraries which were then used for sequencing on the NextSeq 500 platform (Illumina, San Diego, CA, USA) using paired-end 75 bp reagents at the Tayside Centre for Genomic Analysis (University of Dundee).

### Amplicon sequencing

#### Identifying mutations in 400 bp amplicons from multiple meiotic genes

96 DNA samples isolated from three 16 × 16 blocks from the 30 mM EMS treatment (i.e. a total of 768 plants) were used for the following experiment. 400 bp genomic segments from 10 meiotic genes of interest (except for a *HvFANCM* fragment of 479 bp) were identified for PCR amplification from the 96 pooled DNAs (Fig. 1). 400 bp was chosen for compatibility with Illumina MiSeq 2 × 250 paired end sequencing. Gene-specific oligonucleotides were designed with Tm (melting temperature) differences of less than 1 °C for multiplexing using Primer3 (v. 0.4.0) and tagged with 5′-end tails to allow subsequent for sample indexing (Nextera XT) modifying the Illumina protocol for 16S Metagenomic Sequencing Library Preparation (Part # 15044223 Rev. B; custom primers in Additional file 9: Table S8). PCR conditions were optimised by varying template genomic DNA amounts (0–50 ng) and testing different annealing temperatures (60–68 °C) for each primer pair. After optimisation, first round PCR reactions (25 μl) were performed using KAPA HiFi HotStart ReadyMix (Roche), with 25 ng of pooled genomic DNA and 0.2 µM of each primer at 95 °C for 3 min followed by 25 cycles of 95 °C for 30 s, 65 °C for 30 s and 72 °C for 30 s followed by a final extension at 72 °C for 5 min. Amplicons of the expected 400 bp size were verified in 1.6% agarose gels. PCR products were purified with AMPure XP beads (Beckman Coulter Inc.) then indexed using the Nextera XT Index Kit (Illumina). Second round PCR products were cleaned with Agencourt AMPure XP beads, quantified with Quant-iT™ PicoGreen dsDNA Assay Kit, and normalised to 4 nM. The final sequencing library was made by pooling 5 µl of each 96 indexed samples and sequenced on the MiSeq platform (Illumina) using paired-end 250 bp reagents as recommended.

#### Amplicon sequencing from HvMET1A

For the second amplicon sequencing experiment, we used a bigger population of 384 DNA samples isolated from twelve 16 × 16 blocks from the 30 mM EMS treatment (3072 plants). Thirteen regions of 400 bp length were chosen within the *HvMet1A* gene with the same basic reasoning as above. Primers flanking these regions were designed using Primer3 to have a Tm difference of below 1 °C. Primers were tailed at their 5′-end with index-compatible tags as above (Additional file 9: Table S8). PCR conditions were optimized as described above after separating into two reactions to avoid overlapping amplicons. After optimisation, the first PCR amplification was done using Q5 HotStart High Fidelity Taq Polymerase (New England Biolabs, Ipswich, MA, USA), with 25 ng of DNA Template for each pool of individuals and 0.2 µM of each primer. The PCR program was composed of a first denaturation step at 98 °C for 30 s, followed by 35 cycles of: 98 °C for 10 s, 62 °C for 15 s, 72 °C for 45 s, with a final extension at 72 °C for 2 min. Amplicon sizes were checked by running products on 1% agarose gels. Agencourt AmpureXP Beads were used to purify the PCR products before indexing. Primer efficiency during multiplexing was validated by checking that all fragments of interest were amplified equally using qPCR for each pair of primers separately in the presence of 1 μl of 1/5000 dilution of SYBR Green I (Sigma-Aldrich, St. Louis, MI, USA). Indexing using KAPA2G HotStart High Fidelity Taq Polymerase was then done and the products purified using AMPure XP Beads. The products were quantified and diluted to 4 nM before being pooled by combining 5 µl of each of the 96 samples. The Illumina library was quality checked on a Bioanalyzer 2100 (Agilent), quantified using average concentrations of a Qubit Fluorometer and sequenced as above.

### Forward genetics—target exome capture

As part of our forward genetics screen, 274 semi-sterile mutants were identified by close examination of plants in the field. Four M3* seeds from each semi-sterile M2* plant were subsequently sown in the glass house. Lines where semi-sterility was confirmed in the M3* plants were chosen for a targeted exome capture experiment.

#### Target exome capture design

A total of 46 genes were chosen due to their potential impact on meiosis (Additional file 4: Table S3). Based on orthologous searches from *Arabidopsis* and keyword searches in the gene annotation from barley 46 orthologous transcripts were identified and their sequences extracted. The transcripts were blasted against Golden Promise and if needed the coding structure corrected based on annotation from rice (*Oryza sativa*) and Brachypodium. Together with Arbor Biosciences (Ann Arbor, MI, USA), MYbaits were designed for the respective transcripts. We chose a 3×-tiling density and a probe size of 80 nt. Probes which aligned to multiple places in the barley *cv.* Golden Promise reference assembly were removed. This resulted in a total of 4860 probes and a targeted capture array of 189,341 bp.

#### DNA extraction and library preparation

Two seeds for each identified line (M4* generation) were sown and genomic DNA was extracted from 7 to 10 day old leaf material using QIAamp 96 DNA QIAcube HT kit (Qiagen Gmbh) on a QIAcube HT 96 automated nucleic acid purification robot (Qiagen Gmbh). DNA concentration was determined using PicoGreen and adjusted to 10 ng/µl in 1× TE buffer.

#### Sample library preparation

The sample libraries were prepared following the KAPA Hyper Prep Kit (Roche). For each sample, 60 µl of 10 ng/µl DNA was fragmented using a Diagenode Bioruptor (Diagenode, Ougrée, Belgium) aiming for a size range of 180–220 bp (15 min fragmentation (30 s on/30 s off) with a spin after cycle 5 then spin after cycle 10 in 0.1 ml tubes). Quality and concentration were then checked on a Bioanalyzer 2100. Sample library preparation was carried out using the KAPA Hyper Prep Kit as recommended. The KAPA Dual-Indexed Adapter Kit (Roche) was used at 15 µM. An additional library clean-up step was introduced after post-ligation clean up by using a Qiagen MinElute PCR Purification kit (Qiagen Gmbh). A total of 20 µl of each library was amplified in a pre-capture LM-PCR (ligation-mediated PCR, 50 µl; KAPA Hyper Prep Kit (Roche; P5: AATGATACGGCGACCACCGAGA; P7: CAAGCAGAAGACGGCATACGAG; KAPA Hifi Hotstart; Ready mix 2×, 25 µl; Oligo mix 5 µl (500 ng/µl of each oligo); program: 98 °C for 45 s, 9 cycles of 15 s at 98 °C, 30 s at 60 °C and 30 s at 72 °C, followed by 1 min at 72 °C). Libraries were cleaned, and concentrations quantified on a NanoDrop. Quality was again confirmed on a Bioanalyzer 2100, aiming for a library with fragments in the range of 250–500 bp. Library concentrations were normalised to 10 ng/µl and three pools were created using 4 µl of 80, 79 and 78 individual libraries respectively. Pooled libraries were dried using an Eppendorf Concentrator Plus centrifuge (Eppendorf, Stevenage, UK).

#### Hybridization and targeted exome capture

Hybridization and exome capture were carried out using MYcroarray MYbaits In-Solution Sequence Capture for Targeted High-Throughput Sequencing (Manual Version 3.01, Arbor Biosciences, Ann Arbor, MI, USA). A total of 12 µl of ‘LIBs’ mix which included 7 µl of resuspended libraries and 5 µl of block mix were heated to 95 °C for 5 min. ‘LIBs’ mix was then cooled to 65 °C and 18.5 µl of ‘HYBs’ mix which included 12 µl of hybridisation mixes, 5 µl of baits and 1 µl of RNase block was heated to 65 °C for 5 min. Subsequently, 18 µl of ‘HYB’ mix was added to the ‘LIB’ mix and hybridised for 20 h.

#### Washing and recovering captured multiplexed DNA sample

Dynabeads MyOne Streptavidin C1 (Invitrogen, Carlsbad, CA, USA) were prepared with 3 washes using 200 µl of Binding buffer with a final resuspension in 70 µl of binding buffer. This was warmed to 65 °C and mixed with each capture reaction. This mix was incubated at 65 °C for 30 min with mixing every 5 min to keep beads in suspension. Beads were washed three times in 500 µl of wash buffer 2.2 for 10 min at 65 °C with a final resuspension in 30 µl of 10 mM Tris–Cl, 0.05% TWEEN-20 (pH8.0–8.5). A post-capture LM-PCR was done to amplify the bead bound library (Identical to pre-capture LM PCR, except the number of cycles was increased to 14). Agencourt AMPure XP Beads were used to clean up the library which was then resuspended in 10 mM Tris–Cl pH8. 25 µl of supernatant which contains the amplified sample library were transferred into a new tube. A Qubit Fluorometer was used to determine DNA concentration and a Bioanalyzer 2100 to check for the correct fragment size of the final library which were then used for sequencing on the MiSeq platform (Illumina) using paired-end 75 bp reagents as recommended.

### Bioinformatic analyses

#### Whole exome capture

Illumina reads were mapped to the Golden Promise genome reference assembly (Schreiber et al., in prep) using bwa mem (v0.7.17) [57, 58]. The alignment was filtered (alignment score of 70) and sorted using Samtools [59]. MarkDuplicates from the Picard toolset (v.2.18.4) [42] was used to remove duplicates. The variant calling was done using GATK4 (4.0.4) following the best practice advise [60, 61]. For the GATK base quality score recalibration (BQSR) one round of haplotype calling was done, sampling the variants with a quality score of above 30. Calibration was followed by a second and final round of haplotype calling. All 20 datasets were joined by chromosome using GenomicsDBImport and the variants extracted using GenotypeGVCFs. Everything was combined to one vcf file which was filtered using Vcftools [62], SnpSift [63] and GATK’s VariantFiltration for a minimum of 4 reads per sample and sufficient reads in at least 15 out of the 20 samples. Indels were removed and the SNP set was filtered, removing variants with either QD < 2.0 or FS > 20.0 combined with SOR > 4.0. Further filtering involved allowing only one SNP in the 20 plants at any given position.

#### Amplicons

A custom reference was built for both experiments. For the 10-gene experiment, each 400 bp target region was taken and extended by 100 bp on either side. For the single gene experiment, the *HvMet1A* gene sequence was used. Illumina reads were first checked for quality using FastQC (version 0.11.8, http://www.bioinformatics.babraham.ac.uk/projects/fastqc) and afterwards trimmed using Trimmomatic (parameters: Leading 30; Trailing 30; Minlen 100) [64]. Trimmed reads were mapped against the respective references using bwa mem (v0.7.10). The alignment was filtered (minimum alignment score of 220) and sorted using Samtools. Variant calling was done on each of the files separately using Freebayes (v.0.9.18) [43] with relaxed settings: “--haplotype-length 0 --min-alternate-total 30 --min-alternate-fraction 0.02 --pooled-continuous --no-complex --no-mnps --dont-left-align-indels --no-indels --no-population-priors”. Variants were first filtered using Bcftools for individual variants per column per block or per row per block. A SNP was only called as true if it occurred twice in a block: once in a row and once in a column. Reads were also filtered for the expected mutations G > A or C > T. To evaluate the possible effect these mutations might have on the final protein we used PROVEAN (Protein Variation Effect Analyzer, http://provean.jcvi.org/index.php) [65, 66] on a local server with the non-redundant NCBI database (updated October 2018).

#### Targeted exome capture

Illumina reads were first checked for quality using FastQC and trimmed using Trimmomatic (parameters: Leading 10; Trailing 10; Minlen 60). Trimmed reads were mapped against the Golden Promise reference assembly using bwa mem and sorted using Samtools. Variant calling was done using Freebayes with default settings. All output files were merged using Bcftools and then filtered with Vcftools and Vcffilter (https://github.com/vcflib/vcflib). Insertions or deletions were removed and only variants kept which had at least 4 reads as support. In addition, only the variants from the target region were extracted. For the heterozygous sites the alternative allele needed to account for at least 40% of the reads. Again, PROVEAN was used to predict the effect of the variant on the protein.

## Supplementary information

**Additional file 1: Table S1.** Transitions and transversions across the TILLING population from the barley whole exome capture results.

**Additional file 2: Figure S1.** Predicted effects of the identified variants from the barley whole exome capture. Using the barley annotation of the BaRT transcriptome mapped to Golden Promise, SnpEff was used to predict the effect of the variants.

**Additional file 3: Table S2.** Results of the effects as predicted by SnpEff on the individual transcripts.

**Additional file 4: Table S3.** Selected genes from our custom target exome capture. Those genes have been identified as potentially involved in different processes of meiosis.

**Additional file 5: Table S4.** Identified variants from the barley whole exome capture. Variants were identified in 8 genes out of 46 potential meiotic genes across all 20 plants screened.

**Additional file 6: Table S5.** Simultaneous identification of variants from multiple genes. Screening for variants across a 400 bp region in 10 different genes in 768 plants.

**Additional file 7: Table S6.** Identification of variants across the coding sequence of a single gene. Screening for variants in the HvMET1A gene in 3072 plants. Validation was only performed in plants from plate 1 (Plant Names B1, B2 and B3).

**Additional file 8: Table S7.** Identified variants from targeted exome capture on semi-sterile barley *cv.* Golden Promise lines.

**Additional file 9: Table S8.** Primer sequences for targeted amplicon sequencing. Underlined region shows the 5′-end tails for sample indexing.

## Data Availability

The datasets generated and analysed during the current study are available from the NCBI Sequence Read Archive (SRA) (https://www.ncbi.nlm.nih.gov/sra) in the bioproject PRJNA549439.
