# Supplementary material for: A highly mutagenised barley (cv. Golden Promise) TILLING population coupled with strategies for screening-by-sequencing
Source: Plant Methods. 2019 Aug 24;15:99. doi: 10.1186/s13007-019-0486-9 (PMC6708184; doi:10.1186/s13007-019-0486-9)
Supplement: Supplementary file 1 — Additional file 1: Table S1. Transitions and transversions across the TILLING population from the barley whole exome capture results. [file 13007_2019_486_MOESM1_ESM.docx]

**Table S1** Transitions and transversions across the TILLING population from the barley whole exome capture results.

| **Transitions** | Percentage |
| --- | --- |
| G/C -> A/T | 79.4 |
| A/T -> G/C | 3.9 |
| **Transversions** |  |
| A/T -> T/A | 8.1 |
| G/C -> C/G | 0.9 |
| A/G -> C/T | 6.8 |
| C/T -> A/G | 0.9 |
