# Supplementary figures and images for: A highly mutagenised barley (cv. Golden Promise) TILLING population coupled with strategies for screening-by-sequencing
Source: Plant Methods. 2019 Aug 24;15:99. doi: 10.1186/s13007-019-0486-9 (PMC6708184; doi:10.1186/s13007-019-0486-9)

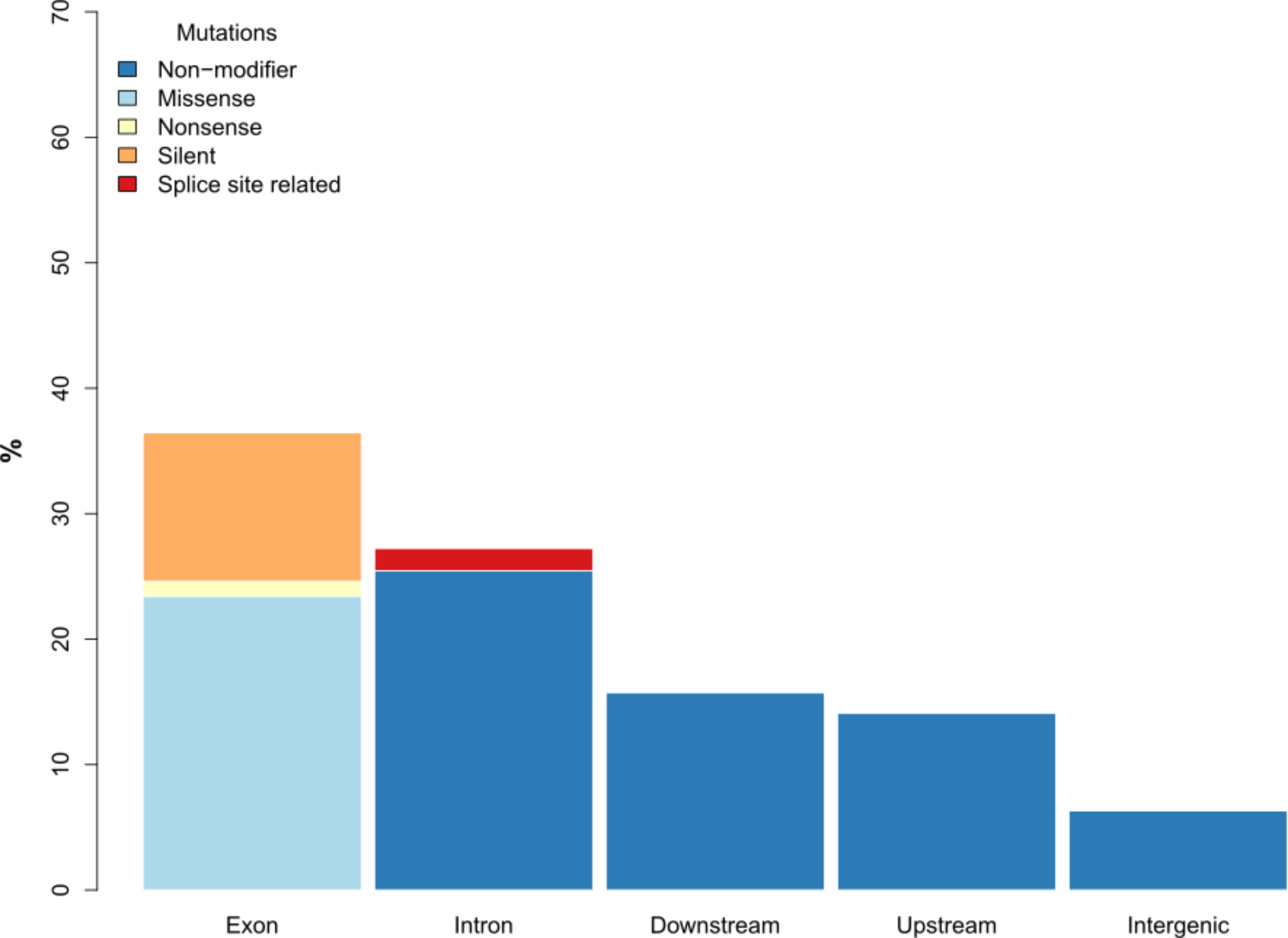

Supplement: Supplementary file 2 — Additional file 2: Figure S1. Predicted effects of the identified variants from the barley whole exome capture. Using the barley annotation of the BaRT transcriptome mapped to Golden Promise, SnpEff was used to predict the effect of the variants. [file 13007_2019_486_MOESM2_ESM.png]
