# Supplementary material for: A highly mutagenised barley (cv. Golden Promise) TILLING population coupled with strategies for screening-by-sequencing
Source: Plant Methods. 2019 Aug 24;15:99. doi: 10.1186/s13007-019-0486-9 (PMC6708184; doi:10.1186/s13007-019-0486-9)
Supplement: Supplementary file 4 — Additional file 4: Table S3. Selected genes from our custom target exome capture. Those genes have been identified as potentially involved in different processes of meiosis. [file 13007_2019_486_MOESM4_ESM.docx]

**Table S3** Selected genes from our custom target exome capture.

| Gene | Barley Identifier | Arabidopsis Orthologue | Rice Orthologue | Brachypodium Orthologue | Name | Predicted Protein Function |
| --- | --- | --- | --- | --- | --- | --- |
| HvASY1 | HORVU5Hr1G076340 | AT1G67370 | LOC_Os09g32930 | Bradi4g34650 | ASYnaptic | Involved in meiotic recombination, more specifically meiotic initiation. Acts as an ASY1 homolog pairing factor |
| HvATM | HORVU3Hr1G000420 | AT3G48190 | LOC_Os01g01689 | Bradi2g00627 | Ataxua-Telangiectasia Mutated | Involved in DNA damage response and acts as a DNA double-strand break response kinase. |
| HvATR | HORVU7Hr1G118750 | AT5G40820 | LOC_Os06g50910 | Bradi1g30007 | Ataxua-Telangiectasia mutated and Rad3-related | Involved in DNA damage response and acts as a DNA single-strand break response kinase. |
| HvAURORA3A | HORVU1Hr1G067200 | AT2G45490 | LOC_Os03g55620 | Bradi1g07590 | Aurora Kinase | Involved in chromatin organisation by histone phosphorylation. Acts as an Aurora kinase |
| HvBRCA1 | HORVU1Hr1G077590 | AT4G21070 | LOC_Os05g43610 | Bradi2g20260 | BReast CAncer susceptibility | Involved in DNA damage response as part of a BRCA1–BARD1 DNA-damage response heterodimer. |
| HvCHD4 | HORVU2Hr1G049320 | AT5G44800 | LOC_Os07g31450 | Bradi1g26940 | Chromodomain-helicase-DNA-binding protein | Involved in chromatin organisation as part of the Snf2-like group. Acts as a Chd3/Mi-2 chromatin remodelling factor |
| HvDDM1A | HORVU4Hr1G008870 | AT5G66750 | LOC_Os03g51230/ LOC_Os09g27060 | Bradi1g10360 | Decreased DNA Methylation | Involved in chromatin organisation by RNA-independent DNA methylation. Acts as a DDM1 chromatin remodelling factor |
| HvDMC1 | HORVU5Hr1G040730 | AT3G22880 | LOC_Os11g04954/ LOC_Os012g04980 | Bradi4g42957 | Disrupted Meiotic cDNA | Involved in meiotic recombination, more specifically in DNA strand exchange. Acts as a recombinase. |
| HvEXO1 | HORVU3Hr1G076270 | AT1G29630 | LOC_Os01g56940 | Bradi2g51637 | EXOnuclease | Involved in DNA damage response, more specifically homologous recombination repair (HR). Acts as an exonuclease. |
| HvFANCM | HORVU4Hr1G018400 | AT1G35530 | LOC_Os11g07870 | Bradi4g23960 | Fanconi anemia complementation group M | Involved in meiotic recombination, more specifically in meiotic crossover as part of the FANCM-MHF DNA remodelling complex. Acts as a FANCM DNA translocase component |
| HvFIGL1 | HORVU5Hr1G029910 | AT3G27130+ AT3G27120 (KM055500) | LOC_Os12g25720 | Bradi4g07862 | Fidgetin-like protein 1 | Involved in DNA damage response, more specifically in double-strand break (DBS) repair via homologous recombination (HR). Acts as an AAA ATPase. |
| HvHEI10 | HORVU6Hr1G040680 | AT1G53490 | LOC_Os02g13810 | Bradi3g08880 | Human Enhancer of Invasion 10 | Involved in meiotic recombination, more specifically in meiotic crossover of the class I interference-sensitive pathway. Acts as an accessory protein. |
| HvHSP90-3 | HORVU5Hr1G072420 | AT5G56000/ AT5G6010/ AT5G6030 | LOC_Os09g30412/ LOC_Os09g30418 |  | Heat Shock Protein 90 | Involved in response to temperature. Is part of the big Hsp (heat-shock-responsive protein) 90 family. |
| HvINO80 | HORVU4Hr1G046140 | AT3G57300 | LOC_Os03g22900 | Bradi1g62320 | INOsitol requiring | Involved in chromatin organisation as part of the Swr1-like group. Acts as an Ino80 chromatin remodelling factor |
| HvISW2A | HORVU1Hr1G021540 | AT3G06400/ AT5G18620 | LOC_Os05g05780 | Bradi2g35740/ Bradi4g09970 | Imitation SWitch subfamily | Involved in chromatin organisation as part of the Snf2-like group. Acts as an Iswi chromatin remodelling factor |
| HvISW2B | HORVU3Hr1G012850 | AT3G06400/ AT5G18620 | LOC_Os01g27040 | Bradi2g12950 | Imitation SWitch subfamily | Involved in chromatin organisation as part of the Snf2-like group. Acts as an Iswi chromatin remodelling factor |
| HvKU70 | HORVU5Hr1G012090 | AT1G16970 | LOC_Os07g08729 | Bradi1g55250 | 70 kDa | Involved in DNA damage response, more specifically nonhomologous end-joining repair (NHEJ). Is part of the Ku70-Ku80 helicase complex. |
| HvKU80 | HORVU0Hr1G038620 | AT1G48050 | LOC_Os03g63920 | Bradi1g01007 | 80 kDa | Involved in DNA damage response, more specifically nonhomologous end-joining repair (NHEJ). Is part of the Ku70-Ku80 helicase complex. |
| HvMER3 | HORVU4Hr1G049830 | AT3G27730 | LOC_Os02g40450 | Bradi3g48850 | MEiotic Recombination | Involved in meiotic recombination, more specifically in meiotic crossover class I interference-sensitive pathway. Acts as a DNA helicase. |
| HvMET1A | HORVU0Hr1G039040 | AT5G49160/ AT4G08990/ AT4G14140/ AT4G13610 | LOC_Os07g08500 | Bradi1g55287 | METhionine requiring | Involved in chromatin organisation by RNA-independent DNA methylation. Acts as a MET DNA methyltransferase. |
| HvMRE11A | HORVU2Hr1G116540 | AT5G54260 | LOC_Os04g54340 | Bradi5g23230 | Meiotic REcombination | Involved in meiotic recombination, more specifically in meiotic double strand break processing as part of the MRE11-RAD50-NBS1 (MRN) complex. |
| HvMSH2 | HORVU1Hr1G030930 | AT3G18524 | LOC_Os05g19270 | Bradi1g15260 | MutS homolog (MutS = Mutator S) | Involved in DNA damage response, more specifically in mismatch repair (MMR) as part of a heterodimer. |
| HvMUS81A | HORVU3Hr1G106210 | AT4G30870 | LOC_Os01g71960 | Bradi2g60640 | MMS and UV Sensitive | Involved in meiotic recombination, more specifically in meiotic crossover class II interference-insensitive pathway. Acts as part of a MUS81-EME1 Holliday junction cleavage heterodimer. |
| HvMUS81B | HORVU3Hr1G039160 | AT4G30870 |  | Bradi2g10500 | MMS and UV Sensitive | Involved in meiotic recombination, more specifically in meiotic crossover class II interference-insensitive pathway. Acts as part of a MUS81-EME1 Holliday junction cleavage heterodimer. |
| HvNBS1 | HORVU1Hr1G043110 | AT3G02680 | LOC_Os10g34580 | Bradi3g29390 | Nijmegen Breakage Syndrome | Involved in meiotic recombination, more specifically in meiotic double strand break processing as part of the MRE11-RAD50-NBS1 (MRN) complex. |
| HvPCH2 | HORVU2Hr1G082720 | AT4G24710 | LOC_Os04g40290 | Bradi5g13467 | Pachytene CHeckpoint | Involved in meiotic recombination, more specifically in meiotic crossover class I interference-sensitive pathway. Acts as a pachytene checkpoint protein. |
| HvPCNA | HORVU6Hr1G088120 | AT2G29570 | LOC_Os02g56130 | Bradi3g54630 | Proliferating Cell Nuclear Antigen | Involved in DNA replication as part of elongation. Acts as a PCNA sliding clamp protein. |
| HvPTB1A | HORVU4Hr1G039070 | AT1G10710 | LOC_Os06g27860 | Bradi1g40622 | Polypyrimidine Tract-Binding | Involved in RNA splicing as part of spliceosome-associated non-snRNP factors. |
| HvRAD17 | HORVU4Hr1G063910 | AT5G66130 | LOC_Os03g13850 | Bradi1g68237 | RADiation sensitive | Involved in sister chromatid separation as part of the spindle assembly checkpoint machinery. |
| HvRAD51 | HORVU7Hr1G091450 | AT5G20850 | LOC_Os12g31370 | Bradi4g07050 | RADiation sensitive | Involved in DNA damage response as part of the BRCC DNA-damage response complex. |
| HvRAD54 | HORVU6Hr1G078680 | AT3G19210 | LOC_Os02g52510 | Bradi3g58092 | RADiation sensitive | Involved in chromatin organisation as part of Rad54-like group. Acts as Rad54 chromatin remodelling factor |
| HvREC8-1 | HORVU1Hr1G062190 | AT5G05490 | LOC_Os05g50410 | Bradi1g27344/ Bradi2g15127 | RECombination | Involved in sister chromatid separation as part of the cohesin regulator complex. Acts as a SCC1 mitotic-specific Kleisin-type component |
| HvRECQL1 | HORVU4Hr1G032190 | AT3G05740 | LOC_Os11g44910 | Bradi4g10110 | RECombination protein Q like | Involved in meiotic recombination, more specifically in meiotic crossover in RTR Holliday junction dissolution complex. Acts as RecQ4A helicase component. |
| HvRECQL4 | HORVU2Hr1G075870 | AT1G10930/ AT1G60930 | LOC_Os04g35420 | Bradi5g10432 | RECombination protein Q like | Involved in meiotic recombination, more specifically in meiotic crossover in RTR Holliday junction dissolution complex. RecQ4A helicase component |
| HvRPA1A | HORVU6Hr1G081140 | AT2G06510 | LOC_Os02g53680 | Bradi1g70030/ Bradi3g57290 | Replication Protein A | Involved in DNA replication as part of elongation. Acts as part of single-stranded-DNA binding RPA1 complex. |
| HvRPA2C | HORVU7Hr1G099210 |  | LOC_Os06g47830 | Bradi1g33430 | Replication Protein A | Involved in DNA replication as part of elongation. Acts as part of single-stranded-DNA binding RPA2 complex. |
| HvRTEL1 | HORVU3Hr1G051380 | AT1G79950 | LOC_Os01g40980 | Bradi2g42410 | Regulator of Telomere ELongation helicase 1 | Involved in DNA damage response, more specifically in telomere maintenance. |
| HvSET1 | HORVU5Hr1G088420 | AT3G61740 | LOC_Os09g38440 | Bradi4g3762 | SET domain-containing | Involved in chromatin organisation via histone lysine methylation/demethylation. Acts as class V/Su(var) histone methyltransferase component |
| HvSPO11-1 | HORVU5Hr1G095640 | AT3G13170 | LOC_Os03g54091 | Bradi1g08390 | Sporulation | Involved in meiotic recombination, more specifically in meiotic double strand break initiation as part of the meiotic topoisomerase-VI complex. |
| HvSPO11-2 | HORVU7Hr1G071060 | AT1G63990 | LOC_Os08g06050 | Bradi3g16580 | Sporulation | Involved in meiotic recombination, more specifically in meiotic double strand break initiation as part of the meiotic topoisomerase-VI complex. |
| HvSPO11-3 | HORVU4Hr1G057310 | AT5G02820 | LOC_Os03g17610 | Bradi1g65870 | Sporulation | Involved in meiotic recombination, more specifically in meiotic double strand break initiation as part of the meiotic topoisomerase-VI complex. |
| HvSR45 | HORVU3Hr1G110470 | AT1G07350 | LOC_Os03g15890 | Bradi1g67145 | Serine/arginine-rich 45 | Involved in RNA processing as part of the RNA quality control Exon Junction complex (EJC). |
| HvSR45A | HORVU7Hr1G122590 | AT1G07350 | LOC_Os08g29650 | Bradi1g29180/ Bradi3g34860 | Serine/arginine-rich 45 | Involved in RNA processing as part of the RNA quality control Exon Junction complex (EJC). |
| HvTOP2A | HORVU6Hr1G067930 | AT3G23890 | LOC_Os02g47150 | Bradi3g52470 | Topoisomerase | Involved in DNA replication, mainly in the preinitiation. Acts as TOP2 DNA topoisomerase |
| HvTOP3α | HORVU4Hr1G074180 | AT5G63920 | LOC_Os03g06900 | Bradi1g74120 | Topoisomerase | Involved in meiotic recombination, more specifically in meiotic crossover as part of the RTR Holliday junction dissolution complex. |
| HvTSN | HORVU1Hr1G037340 | AT2G37020 | LOC_Os10g30640 | Bradi3g27660 | Translin | DNA-binding protein that specifically recognizes consensus sequences at the breakpoint junctions in chromosomal translocations |

Those genes have been identified as potentially involved in different processes of meiosis.
