# Supplementary material for: A highly mutagenised barley (cv. Golden Promise) TILLING population coupled with strategies for screening-by-sequencing
Source: Plant Methods. 2019 Aug 24;15:99. doi: 10.1186/s13007-019-0486-9 (PMC6708184; doi:10.1186/s13007-019-0486-9)
Supplement: Supplementary file 5 — Additional file 5: Table S4. Identified variants from the barley whole exome capture. Variants were identified in 8 genes out of 46 potential meiotic genes across all 20 plants screened. [file 13007_2019_486_MOESM5_ESM.docx]

**Table S4** Identified variants from the barley whole exome capture.

| **Plant** | **Gene** | **Ref** | **Alt** | **Nt pos** | **Aa ref** | **Aa alt** | **Aa pos** | **Aa effect** | **Provean Score** | **Zygosity (pred.)** | **Depth (Ref, Alt)** |
| --- | --- | --- | --- | --- | --- | --- | --- | --- | --- | --- | --- |
| 2018_S11 | HvCHD4 | G | A | 1977 | Arg | Arg | 659 | Synonymous | 0 | Hom | 0,7 |
| 2016_S6 | HvCHD4 | G | A | 1163 | Pro | Leu | 388 | Nonsynonymous | -2.293 | Hom | 0,7 |
| 2018_S6 | HvCHD4 | G | A | 650 | Ser | Phe | 217 | Nonsynonymous | -2.254 | Hom | 0,6 |
| 2018_S5 | HvMET1A | C | T | 2049 | Glu | Glu | 683 | Synonymous | 0 | Hom | 0,9 |
| 2016_S2 | HvMRE11 | G | A | 289 | Pro | Ser | 97 | Nonsynonymous | -1.519 | Het | 2,12 |
| 2016_S7 | HvMRE11 | G | A | 228 | Arg | Arg | 76 | Synonymous | 0 | Het | 5,9 |
| 2016_S5 | HvMUS81B | G | A | 195 | Lys | Lys | 65 | Synonymous | 0 | Hom | 0,20 |
| 2018_S5 | HvMUS81B | C | T | 1081 | Pro | Ser | 361 | Nonsynonymous | -5.909 | Het | 32,22 |
| 2016_S2 | HvSR45 | C | T | 571 | Pro | Ser | 191 | Nonsynonymous | -6.485 | Hom | 0,16 |
| 2018_S8 | HvDDM1A | G | A | 18 | His | His | 6 | Synonymous | 0 | Het | 3,4 |
| 2016_S7 | HvPTB1 | A | G | 1264 | Thr | Ala | 422 | Nonsynonymous | 0.028 | Het | 9,8 |
| 2018_S5 | HvTOP3α | G | A | 2902 | Pro | Ser | 968 | nonsynonymous | -5.581 | Hom | 0,16 |

Variants were identified in 8 genes out of 46 potential meiotic genes across all 20 plants screened.
