# Supplementary material for: A highly mutagenised barley (cv. Golden Promise) TILLING population coupled with strategies for screening-by-sequencing
Source: Plant Methods. 2019 Aug 24;15:99. doi: 10.1186/s13007-019-0486-9 (PMC6708184; doi:10.1186/s13007-019-0486-9)
Supplement: Supplementary file 6 — Additional file 6: Table S5. Simultaneous identification of variants from multiple genes. Screening for variants across a 400 bp region in 10 different genes in 768 plants. [file 13007_2019_486_MOESM6_ESM.docx]

**Table S5** Simultaneous identification of variants from multiple genes.

| **Plant No.** | **Gene** | **Ref** | **Alt** | **Nt pos** | **Aa ref** | **Aa alt** | **Aa pos** | **Aa effect** | **Provean Score** | **Zygosity (pred.)** | **Validation**  **WT:Het:Mut** |
| --- | --- | --- | --- | --- | --- | --- | --- | --- | --- | --- | --- |
| B1C1RE | HvRAD54 | C | T | 369 | Ile | Ile | 123 | Synonymous | 0 | Het | No germ. |
| B1C2RD | HvRAD54 | G | A | 593 | Gly | Asp | 198 | Nonsynonymous | -5.002 | Het | 2:1:2 |
| B1C2RJ | HvPCH2 | G | A | Intron | - | - | - | - | - | Het | 3:0:4 |
| B1C2RL | HvFANCM | G | A | 2034 | Gln | Gln | 678 | Synonymous | 0 | Het | sterile |
| B1C4RD | HvMET1A | C | T | 743 | Thr | Ile | 248 | Nonsynonymous | -1.503 | Hom | 0:0:6 |
| B1C4RJ | HvFANCM | G | A | 2052 | Gly | Gly | 684 | Synonymous | 0 | Het | 4:4:0 |
| B1C8RL | HvDDM1A | C | T | 320 | Ala | Val | 107 | Nonsynonymous | -2.756 | Hom | 0:0:7 |
| B1C15RI | HvREC8 | C | T | Intron | - | - | - | - | - | Hom | No germ. |
| B1C16RJ | HvTOPIIA | G | A | 838 | Glu | Lys | 280 | Nonsynonymous | 0 | Het | No germ. |
| B2C1RM | HvMET1A | G | A | 685 | Glu | Lys | 229 | Nonsynonymous | -3.402 | Het | No germ. |
| B2C3RO | HvTOPIIA | G | A | 838 | Glu | Lys | 280 | Nonsynonymous | 0 | Het | 0:1:2 |
| B2C6RN | HvTOPIIA | G | A | 724 | Val | Ile | 242 | Nonsynonymous | -0.269 | Hom | sterile |
| B2C14RO | HvREC8 | C | T | Intron | - | - | - | - | - | Hom | No germ. |
| B3C1RM | HvREC8 | C | T | Intron | - | - | - | - | - | Het | 1:2:0 |
| B3C2RM | HvTOPIIA | G | A | 810 | Val | Val | 270 | Synonymous | 0 | Het | No germ. |
| B3C10RJ | HvDDM1A | C | T | 320 | Ala | Val | 107 | Nonsynonymous | -2.756 | Het | 0:0:2 |
| B3C11RB | HvREC8 | C | T | Intron | - | - | - | - | - | Hom | sterile |

Screening for variants across a 400bp region in 10 different genes in 768 plants.
