# Supplementary material for: A highly mutagenised barley (cv. Golden Promise) TILLING population coupled with strategies for screening-by-sequencing
Source: Plant Methods. 2019 Aug 24;15:99. doi: 10.1186/s13007-019-0486-9 (PMC6708184; doi:10.1186/s13007-019-0486-9)
Supplement: Supplementary file 7 — Additional file 7: Table S6. Identification of variants across the coding sequence of a single gene. Screening for variants in the HvMET1A gene in 3072 plants. Validation was only performed in plants from plate 1 (Plant Names B1, B2 and B3). [file 13007_2019_486_MOESM7_ESM.docx]

**Table S6** Identification of variants across the coding sequence of a single gene.

| **Plant Name** | **Ref** | **Alt** | **Nt pos** | **Aa ref** | **Aa alt** | **Aa pos** | **Aa effect** | **Provean Score** | **Zygosity (pred.)** | **Validation**  **WT:Het:Mut** |
| --- | --- | --- | --- | --- | --- | --- | --- | --- | --- | --- |
| B1C10RE | G | A | 2520 | Glu | Glu | 840 | Synonymous | 0 | Hom | 0:0:5 |
| B1C10RJ | G | A | Intron | - | - | - | - | - | Het | 0:1:2 |
| B1C13RK | G | A | 2795 | Gly | Asp | 932 | Nonsynonymous | -1.412 | Het | 2:1:1 |
| B2C1RG | G | A | 2547 | Lys | Lys | 849 | Synonymous | 0 | Het | 0:0:7 |
| B2C4RM | G | A | 3967 | Ala | Thr | 1323 | Nonsynonymous | -3.993 | Het | 0:1:5 |
| B2C8RB | C | T | 2164 | Pro | Ser | 722 | Nonsynonymous | -3.007 | Het | sterile |
| B2C13RM | G | A | 3977 | Ser | Asn | 1326 | Nonsynonymous | -2.995 | Hom | 0:0:8 |
| B2C16RN | C | T | Intron | - | - | - | - | - | Het | 4:0:3 |
| B3C3RO | G | A | 3358 | Ala | Thr | 1120 | Nonsynonymous | -3.887 | Het | 2:2:1 |
| B3C5RP | G | A | 2884 | Val | Ile | 962 | Nonsynonymous | -0.718 | Het | 3:0:0 |
| B3C9RA | C | T | 2982 | Tyr | Tyr | 994 | Synonymous | 0 | Het | sterile |
| B3C12RF | G | A | 3977 | Ser | Asn | 1326 | Nonsynonymous | -2.995 | Het | 0:0:2 |
| B3C14RH | C | T | 2164 | Pro | Ser | 722 | Nonsynonymous | -3.007 | Het | - |
| B3C16RG | C | T | 3994 | Pro | Ser | 1332 | Nonsynonymous | -7.620 | Het | 4:0:3 |
| B5C1RO | C | T | 3518 | Ala | Val | 1173 | Nonsynonymous | -3.544 | Het | - |
| B5C8RD | C | T | 2618 | Pro | Leu | 873 | Nonsynonymous | -8.622 | Het | - |
| B6C9RF | C | T | 2618 | Pro | Leu | 873 | Nonsynonymous | -8.622 | Hom | - |
| B7C3RB | C | T | 2618 | Pro | Leu | 873 | Nonsynonymous | -8.622 | Hom | - |
| B7C9RP | C | T | 4130 | Ala | Val | 1377 | Nonsynonymous | 0.976 | Hom | - |
| B7C15RL | C | T | 4295 | Ala | Val | 1432 | Nonsynonymous | -3.757 | Het | - |
| B8C1RP | C | T | 3131 | Ala | Val | 1044 | Nonsynonymous | -2.842 | Het | - |
| B8C12RJ | C | T | 4285 | Pro | Ser | 1429 | Nonsynonymous | -7.781 | Hom | - |
| B9C7RB | C | T | 2618 | Pro | Leu | 873 | Nonsynonymous | -8.622 | Hom | - |
| B10C11RD | C | T | 4295 | Ala | Val | 1432 | Nonsynonymous | -3.757 | Het | - |
| B11C1RA | C | T | 2618 | Pro | Leu | 873 | Nonsynonymous | -8.622 | Hom | - |
| B11C1RL | C | T | Intron | - | - | - | - | - | Het | - |
| B11C10RE | C | T | 2870 | Ala | Val | 957 | Nonsynonymous | -1.430 | Het | - |
| B12C1RB | C | T | 4401 | Cys | Cys | 1467 | Synonymous | 0 | Hom | - |
| B12C3RL | G | A | 198 | Lys | Lys | 66 | Synonymous | 0 | Hom | - |
| B12C9RP | C | T | 3904 | Pro | Ser | 1302 | Nonsynonymous | -7.811 | Hom | - |

Screening for variants in the *HvMET1A* gene in 3072 plants. Validation was only performed in plants from plate 1 (Plant Names B1, B2 and B3).
