# Supplementary material for: A highly mutagenised barley (cv. Golden Promise) TILLING population coupled with strategies for screening-by-sequencing
Source: Plant Methods. 2019 Aug 24;15:99. doi: 10.1186/s13007-019-0486-9 (PMC6708184; doi:10.1186/s13007-019-0486-9)
Supplement: Supplementary file 8 — Additional file 8: Table S7. Identified variants from targeted exome capture on semi-sterile barley cv. Golden Promise lines. [file 13007_2019_486_MOESM8_ESM.docx]

**Table S7** Identified variants from targeted exome capture on semi-sterile barley *cv.* Golden Promise lines.

| Line | Gene | Ref | Alt | Nt pos | Aa ref | Aa alt | Aa pos | Aa effect | Provean Score | Zygosity (pred.) |
| --- | --- | --- | --- | --- | --- | --- | --- | --- | --- | --- |
| **IP-048** | HvATM | G | A | spliceSite | - | - | - | - | - | Hom |
| **IP-172** | HvATM | C | T | 8538 | Arg | Arg | 2846 | synonymous | 0 | Hom |
| **IP-048** | HvATM | G | A | intron | - | - | - | - | - | Hom |
| **IP-005** | HvATR | G | A | 6706 | Pro | Ser | 2236 | nonsynonymous | -7.225 | Hom |
| **IP-116** | HvATR | C | T | 5770 | Glu | Lys | 1924 | nonsynonymous | -3.987 | Hom |
| **IP-169** | HvBRCA1 | G | A | 2267 | Trp | * | 756 | nonsense | Stop codon | Hom |
| **IP-218** | HvBRCA1 | C | T | 2901 | Ala | Ala | 967 | synonymous | 0 | Hom |
| **IP-047** | HvCHD4 | G | A | 1339 | Glu | Lys | 447 | nonsynonymous | -0.663 | Hom |
| **IP-110** | HvCHD4 | G | A | 2055 | Leu | Leu | 685 | synonymous | 0 | Hom |
| **IP-239** | HvDDM1A | G | T | 459 | Lys | Asn | 153 | nonsynonymous | 1.533 | Hom |
| **IP-176** | HvDDM1A | C | T | 1406 | Thr | Ile | 469 | nonsynonymous | -0.287 | Hom |
| **IP-076** | HvDMC1 | G | A | 819 | Asn | Asn | 273 | synonymous | 0 | Hom |
| **IP-086** | HvDMC1 | G | A | intron | - | - | - | - | - | Hom |
| **IP-099** | HvEXO1A | C | T | 2003 | Thr | Ile | 668 | nonsynonymous | -1.805 | Hom |
| **IP-239** | HvEXO1A | C | T | 2687 | Ala | Val | 896 | nonsynonymous | -0.763 | Hom |
| **IP-169** | HvFANCM | G | A | 4193 | Thr | Ile | 1398 | nonsynonymous | -2.403 | Hom |
| **IP-116** | HvFANCM | G | A | 4133 | Ala | Val | 1378 | nonsynonymous | -1.484 | Hom |
| **IP-106** | HvFANCM | G | A | intron | - | - | - | - | - | Hom |
| **IP-234** | HvHEI10 | T | C | 887 | Asn | Ser | 296 | nonsynonymous | -1.104 | Hom |
| **IP-111** | HvHEI10 | C | T | 879 | Gln | Gln | 293 | synonymous | 0 | Hom |
| **IP-130** | HvHSP90-3 | G | A | 1074 | Val | Val | 358 | synonymous | 0 | Hom |
| **IP-130** | HvHSP90-3 | G | A | 966 | Phe | Phe | 322 | synonymous | 0 | Hom |
| **IP-158** | HvHSP90-3 | G | A | 612 | Ser | Ser | 204 | synonymous | 0 | Hom |
| **IP-052** | HvHSP90-3 | C | T | 549 | Gln | Gln | 183 | synonymous | 0 | Hom |
| **IP-204** | HvHSP90-3 | C | T | 549 | Gln | Gln | 183 | synonymous | 0 | Hom |
| **IP-153** | HvINO80 | G | A | 1592 | Gly | Asp | 531 | nonsynonymous | 2.11 | Het |
| **IP-158** | HvINO80 | C | T | 285 | Asn | Asn | 95 | synonymous | 0 | Hom |
| **IP-154** | HvISW2A | C | T | intron | - | - | - | - | - | Hom |
| **IP-238** | HvISW2B | A | T | 2108 | Lys | Ile | 703 | nonsynonymous | -6.783 | Hom |
| **IP-128** | HvISW2B | G | A | 1917 | Glu | Glu | 639 | synonymous | 0 | Hom |
| **IP-155** | HvISW2B | G | A | 2475 | Lys | Lys | 825 | synonymous | 0 | Hom |
| **IP-013** | HvISW2B | C | T | intron | - | - | - | - | - | Hom |
| **IP-130** | HvISW2B | C | T | intron | - | - | - | - | - | Hom |
| **IP-156** | HvISW2B | C | T | intron | - | - | - | - | - | Hom |
| **IP-130** | HvISW2B | T | C | intron | - | - | - | - | - | Hom |
| **IP-156** | HvISW2B | T | C | intron | - | - | - | - | - | Hom |
| **IP-095** | HvISW2B | C | T | intron | - | - | - | - | - | Het |
| **IP-108** | HvKU80 | G | A | 1388 | Ser | Asn | 463 | nonsynonymous | 1.069 | Hom |
| **IP-109** | HvKU80 | G | A | 1388 | Ser | Asn | 463 | nonsynonymous | 1.069 | Hom |
| **IP-187** | HvMER3 | C | A | 92 | Ser | * | 31 | nonsense | Stop codon | Het |
| **IP-086** | HvMER3 | G | A | 167 | Gly | Asp | 56 | nonsynonymous | -6.02 | Hom |
| **IP-053** | HvMER3 | C | T | intron | - | - | - | - | - | Het |
| **IP-153** | HvMET1A | C | T | 316 | Pro | Ser | 106 | nonsynonymous | -6.802 | Hom |
| **IP-174** | HvMET1A | C | T | 670 | Pro | Ser | 224 | nonsynonymous | -6.57 | Hom |
| **IP-086** | HvMET1A | G | A | 3763 | Ala | Thr | 1255 | nonsynonymous | 3.588 | Hom |
| **IP-200** | HvMET1A | G | A | 3977 | Ser | Asn | 1326 | nonsynonymous | -2.951 | Het |
| **IP-013** | HvMRE11A | C | T | 1550 | Ser | Asn | 517 | nonsynonymous | -1.961 | Het |
| **IP-219** | HvMRE11A | G | A | 1423 | Leu | Leu | 475 | synonymous | 0 | Hom |
| **IP-275** | HvMSH2 | G | A | 1477 | His | Tyr | 493 | nonsynonymous | -2.3 | Hom |
| **IP-178** | HvMSH2 | G | A | 22 | Pro | Ser | 8 | nonsynonymous | -0.635 | Hom |
| **IP-214** | HvMSH2 | G | A | 22 | Pro | Ser | 8 | nonsynonymous | -0.635 | Hom |
| **IP-233** | HvMSH2 | G | A | 22 | Pro | Ser | 8 | nonsynonymous | -0.635 | Hom |
| **IP-098** | HvMUS81A | G | A | 196 | Asp | Asn | 66 | nonsynonymous | -3.661 | Het |
| **IP-033** | HvMUS81A | C | T | 1250 | Thr | Ile | 417 | nonsynonymous | -1.463 | Het |
| **IP-044** | HvMUS81A | C | T | intron | - | - | - | - | - | Hom |
| **IP-188** | HvMUS81A | C | T | intron | - | - | - | - | - | Hom |
| **IP-072** | HvNBS1 | A | T | 1573 | Ser | Cys | 525 | nonsynonymous | -4.772 | Hom |
| **IP-255** | HvNBS1 | G | A | 136 | Ala | Thr | 46 | nonsynonymous | -3.416 | Hom |
| **IP-278** | HvNBS1 | G | A | 136 | Ala | Thr | 46 | nonsynonymous | -3.416 | Hom |
| **IP-097** | HvNBS1 | G | A | 1207 | Glu | Lys | 403 | nonsynonymous | -0.422 | Hom |
| **IP-098** | HvNBS1 | G | A | intron | - | - | - | - | - | Hom |
| **IP-100** | HvNBS1 | G | A | intron | - | - | - | - | - | Hom |
| **IP-044** | HvPCH2 | C | T | 659 | Pro | Leu | 220 | nonsynonymous | -9.193 | Hom |
| **IP-094** | HvRAD17 | C | T | intron | - | - | - | - | - | Het |
| **IP-127** | HvRAD51 | G | A | 536 | Ala | Val | 179 | nonsynonymous | -3.749 | Hom |
| **IP-108** | HvRAD51 | G | A | intron | - | - | - | - | - | Hom |
| **IP-109** | HvRAD51 | G | A | intron | - | - | - | - | - | Hom |
| **IP-004** | HvRAD54 | C | T | 823 | Leu | Leu | 275 | synonymous | 0 | Hom |
| **IP-124** | HvRAD54 | C | T | 1617 | Ile | Ile | 539 | synonymous | 0 | Hom |
| **IP-240** | HvRAD54 | C | T | intron | - | - | - | - | - | Hom |
| **IP-084** | HvREC8 | C | T | 1736 | Ala | Val | 579 | nonsynonymous | -3.202 | Het |
| **IP-198** | HvRECQL1 | G | A | 1105 | Ala | Thr | 369 | nonsynonymous | -3.939 | Het |
| **IP-113** | HvRECQL1 | G | A | 266 | Gly | Glu | 89 | nonsynonymous | 0.47 | Het |
| **IP-238** | HvRECQL4 | G | A | 1129 | Pro | Ser | 377 | nonsynonymous | -2.416 | Hom |
| **IP-158** | HvRECQL4 | G | A | 3407 | Ala | Val | 1136 | nonsynonymous | -1.013 | Hom |
| **IP-183** | HvRECQL4 | C | T | 767 | Ser | Asn | 256 | nonsynonymous | -0.254 | Hom |
| **IP-130** | HvSET1 | G | C | 229 | Ala | Pro | 77 | nonsynonymous | 2.635 | Hom |
| **IP-156** | HvSET1 | G | C | 229 | Ala | Pro | 77 | nonsynonymous | 2.635 | Het |
| **IP-130** | HvSET1 | A | G | 919 | Lys | Glu | 307 | nonsynonymous | 1.786 | Hom |
| **IP-156** | HvSET1 | A | G | 919 | Lys | Glu | 307 | nonsynonymous | 1.786 | Het |
| **IP-054** | HvSET1 | G | A | 2737 | Ala | Thr | 913 | nonsynonymous | -1.117 | Het |
| **IP-199** | HvSET1 | G | A | 2737 | Ala | Thr | 913 | nonsynonymous | -1.117 | Hom |
| **IP-130** | HvSET1 | G | A | 980 | Arg | Lys | 327 | nonsynonymous | -0.196 | Hom |
| **IP-156** | HvSET1 | G | A | 980 | Arg | Lys | 327 | nonsynonymous | -0.196 | Het |
| **IP-054** | HvSET1 | G | A | 2118 | Glu | Glu | 706 | synonymous | 0 | Hom |
| **IP-199** | HvSET1 | G | A | 2118 | Glu | Glu | 706 | synonymous | 0 | Hom |
| **IP-094** | HvSET1 | G | A | 3369 | Arg | Arg | 1123 | synonymous | 0 | Hom |
| **IP-188** | HvSPO11-1 | C | T | intron | - | - | - | - | - | Hom |
| **IP-001** | HvSPO11-2 | C | T | 1102 | Leu | Phe | 368 | nonsynonymous | -3.47 | Hom |
| **IP-137** | HvSPO11-2 | A | G | 1037 | Gln | Arg | 346 | nonsynonymous | -3.159 | Het |
| **IP-110** | HvSR45 | C | T | intron | - | - | - | - | - | Hom |
| **IP-048** | HvTOP2A | T | G | 3242 | Asn | Thr | 1081 | nonsynonymous | -3.95 | Hom |
| **IP-238** | HvTOP2A | G | A | 830 | Ala | Val | 277 | nonsynonymous | -3.191 | Hom |
| **IP-187** | HvTOP2A | G | A | 3542 | Ala | Val | 1181 | nonsynonymous | -2.868 | Hom |
| **IP-137** | HvTOP2A | C | A | 3256 | Ala | Ser | 1086 | nonsynonymous | -2.429 | Hom |
| **IP-211** | HvTOP3α | T | A | 2371 | Arg | * | 791 | nonsense | Stop codon | Het |
| **IP-208** | HvTOP3α | G | A | 2017 | Leu | Leu | 673 | synonymous | 0 | Hom |
| **IP-093** | HvTNS | T | C | 198 | Pro | Pro | 66 | synonymous | 0 | Het |
