# Supplementary material for: A highly mutagenised barley (cv. Golden Promise) TILLING population coupled with strategies for screening-by-sequencing
Source: Plant Methods. 2019 Aug 24;15:99. doi: 10.1186/s13007-019-0486-9 (PMC6708184; doi:10.1186/s13007-019-0486-9)
Supplement: Supplementary file 9 — Additional file 9: Table S8. Primer sequences for targeted amplicon sequencing. Underlined region shows the 5′-end tails for sample indexing. [file 13007_2019_486_MOESM9_ESM.docx]

**Table S8** Primer sequences for targeted amplicon sequencing.

| **Oligoname** | **Sequence** |
| --- | --- |
| TP_FIGL1F1 | TCGTCGGCAGCGTCAGATGTGTATAAGAGACAGCCCAGCTTGTGAAAAGTTCACGATT |
| TP_FIGL1R1 | GTCTCGTGGGCTCGGAGATGTGTATAAGAGACAGCCTAGCAGTCACAAATCCATTGGC |
| TP_FANCMF1 | TCGTCGGCAGCGTCAGATGTGTATAAGAGACAGGCAGGTGACCGACGGAAGG |
| TP_FANCMR1 | GTCTCGTGGGCTCGGAGATGTGTATAAGAGACAGTTCAAAGGCGTTCCCTTTACGCA |
| TP_Rad54F1 | TCGTCGGCAGCGTCAGATGTGTATAAGAGACAGTTTTTCCTGGTTTCTGTCAGGGGA |
| TP_Rad54R1 | GTCTCGTGGGCTCGGAGATGTGTATAAGAGACAGACCTTTGATGAGGACGAAGGTAACG |
| TP_Pch2F1 | TCGTCGGCAGCGTCAGATGTGTATAAGAGACAGGGTCCTCGTGTCAGGTAGGCG |
| TP_Pch2R1 | GTCTCGTGGGCTCGGAGATGTGTATAAGAGACAGAAACATACCCAGCAAACTCCACTGC |
| TP_Rad17F1 | TCGTCGGCAGCGTCAGATGTGTATAAGAGACAGTGCTTCAAGGTGCTGGTGCTCA |
| TP_Rad17R1 | GTCTCGTGGGCTCGGAGATGTGTATAAGAGACAGACAGTCCTGTGCTGGAGCTAGAGT |
| TP_RecQl4F1 | TCGTCGGCAGCGTCAGATGTGTATAAGAGACAGGCTGCAGAAGGGCATCGACC |
| TP_RecQl4R1 | GTCTCGTGGGCTCGGAGATGTGTATAAGAGACAGCTCGCTGATTTGGGCGGAAAGA |
| TP_Met1AF1 | TCGTCGGCAGCGTCAGATGTGTATAAGAGACAGAAGGATTTGGGCGTATTGAGGACTG |
| TP_Met1AR1 | GTCTCGTGGGCTCGGAGATGTGTATAAGAGACAGTCTCTTAGAGCAAGAAGAACCGGCA |
| TP_DDM1AF1 | TCGTCGGCAGCGTCAGATGTGTATAAGAGACAGCCATCGACGGGGCGCTGAA |
| TP_DDM1AR1 | GTCTCGTGGGCTCGGAGATGTGTATAAGAGACAGCGGCTTAACCTACAGGCCACACC |
| TP_Rec8F1 | TCGTCGGCAGCGTCAGATGTGTATAAGAGACAGTCCTCGCCGGATTTGCTCTCC |
| TP_Rec8R1 | GTCTCGTGGGCTCGGAGATGTGTATAAGAGACAGCGGCCGGCCTGATCTTCCAT |
| TP_TopIIAF1 | TCGTCGGCAGCGTCAGATGTGTATAAGAGACAGTGGGGAAGAAGTCGGAGCCT |
| TP_TopIIAR1 | GTCTCGTGGGCTCGGAGATGTGTATAAGAGACAGACAGACCTCCCAACGATCATTTACC |
| Met1A_CD01b_F | TCGTCGGCAGCGTCAGATGTGTATAAGAGACAGAGCAAAGAAATGAGATGGAAGGGTGAA |
| Met1A_CD01b_R | GTCTCGTGGGCTCGGAGATGTGTATAAGAGACAGACTTTGGCCCGCTCAACCTTC |
| Met1A_CD02_F | TCGTCGGCAGCGTCAGATGTGTATAAGAGACAGAGAGTTGGTGTCTGTCAATCTCCGA |
| Met1A_CD02_R | GTCTCGTGGGCTCGGAGATGTGTATAAGAGACAGCAGGCCAATGTTTCTTCCTGCCT |
| Met1A_CD03_F | TCGTCGGCAGCGTCAGATGTGTATAAGAGACAGGTCCATGACTTCTTATACATCAGGCCTG |
| Met1A_CD03_R | GTCTCGTGGGCTCGGAGATGTGTATAAGAGACAGATCCACAGGCACATTAATTACATCTTCACT |
| Met1A_CD04_F | TCGTCGGCAGCGTCAGATGTGTATAAGAGACAGTGTCACCATCACTAGTTCCATGCTTG |
| Met1A_CD04_R | GTCTCGTGGGCTCGGAGATGTGTATAAGAGACAGCACCAGCTGCCTTCCGAGC |
| Met1A_CD05_F | TCGTCGGCAGCGTCAGATGTGTATAAGAGACAGTCAGGCAAATGGATGGAGGTGC |
| Met1A_CD05_R | GTCTCGTGGGCTCGGAGATGTGTATAAGAGACAGTCCCCACATTTGTCCATGATTGCC |
| Met1A_CD06_F | TCGTCGGCAGCGTCAGATGTGTATAAGAGACAGACTGCAATGTGATCTTGAAGTAGGTGAA |
| Met1A_CD06_R | GTCTCGTGGGCTCGGAGATGTGTATAAGAGACAGAGGAATGCTAAAATCATCTCACACTGAACT |
| Met1A_CD07_F | TCGTCGGCAGCGTCAGATGTGTATAAGAGACAGTGGTGGCCCTCCCTGTCAG |
| Met1A_CD07_R | GTCTCGTGGGCTCGGAGATGTGTATAAGAGACAGCCTGCTTCTAAGATTCCAAAACGAACCT |
| Met1A_CD08_F | TCGTCGGCAGCGTCAGATGTGTATAAGAGACAGGGTTACCAGGTGCTAATTCTTGCCA |
| Met1A_CD08_R | GTCTCGTGGGCTCGGAGATGTGTATAAGAGACAGAAAACTGAATGGCCTTGGAATATTACCTCA |
| Met1A_CD09_F | TCGTCGGCAGCGTCAGATGTGTATAAGAGACAGCGAAAGTGGAAAATGGTGCAAGTAAACT |
| Met1A_CD09_R | GTCTCGTGGGCTCGGAGATGTGTATAAGAGACAGGTCCACCATTTGCCCAGAAGATAGC |
| Met1A_CD10_F | TCGTCGGCAGCGTCAGATGTGTATAAGAGACAGGGTGTTTGTGAGATGTTAATTCCGGGA |
| Met1A_CD10_R | GTCTCGTGGGCTCGGAGATGTGTATAAGAGACAGTGCCCGCAAACTGGTAGCTG |
| Met1A_CD11_F | TCGTCGGCAGCGTCAGATGTGTATAAGAGACAGGGGAGGTTAGATTGGGAGGGCA |
| Met1A_CD11_R | GTCTCGTGGGCTCGGAGATGTGTATAAGAGACAGCCCTTGGGAGTTCTACAGACGCA |
| Met1A_IE01_F | TCGTCGGCAGCGTCAGATGTGTATAAGAGACAGGCTGGCCACCTCTCATGCC |
| Met1A_IE01_R | GTCTCGTGGGCTCGGAGATGTGTATAAGAGACAGCCTCTGGCCCTAATTTTGTCAGGTT |
| Met1A_IE02_F | TCGTCGGCAGCGTCAGATGTGTATAAGAGACAGTGATAACCAGGATGCAGGAGTTCC |
| Met1A_IE02_R | GTCTCGTGGGCTCGGAGATGTGTATAAGAGACAGAGTACAGGATCACACCAAGGAGCA |

Underlined region shows the 5’-end tails for sample indexing.
